# Identification of the genes involved in odorant reception and detection in the palm weevil *Rhynchophorus ferrugineus*, an important quarantine pest, by antennal transcriptome analysis

**DOI:** 10.1186/s12864-016-2362-6

**Published:** 2016-01-22

**Authors:** Binu Antony, Alan Soffan, Jernej Jakše, Mahmoud M. Abdelazim, Saleh A. Aldosari, Abdulrahman S. Aldawood, Arnab Pain

**Affiliations:** Department of Plant Protection, Chair of Date Palm Research, King Saud University, 11451 Riyadh, Saudi Arabia; Department of Plant Protection, King Saud University, EERU, Riyadh, Saudi Arabia; Biotechnical Faculty, Agronomy Department, University of Ljubljana, SI-1000 Ljubljana, Slovenia; BASE Division, KAUST, Thuwal, Jeddah, 23955-6900 Saudi Arabia

**Keywords:** Red palm weevil, Antennal transcriptome, Chemical communication, Olfactory gene families

## Abstract

**Background:**

The Red Palm Weevil (RPW) *Rhynchophorus ferrugineus* (Oliver) is one of the most damaging invasive insect species in the world. This weevil is highly specialized to thrive in adverse desert climates, and it causes major economic losses due to its effects on palm trees around the world. RPWs locate palm trees by means of plant volatile cues and use an aggregation pheromone to coordinate a mass-attack. Here we report on the high throughput sequencing of the RPW antennal transcriptome and present a description of the highly expressed chemosensory gene families.

**Results:**

Deep sequencing and assembly of the RPW antennal transcriptome yielded 35,667 transcripts with an average length of 857 bp and identified a large number of highly expressed transcripts of odorant binding proteins (OBPs), chemosensory proteins (CSPs), odorant receptors/co-receptors (ORs/Orcos), sensory neuron membrane proteins (SNMPs), gustatory receptors (GRs) and ionotropic receptors (IRs). In total, 38 OBPs, 12 CSPs, 76 ORs, 1 Orco, 6 SNMPs, 15 GRs and 10 IRs were annotated in the *R. ferrugineus* antennal transcriptome. A comparative transcriptome analysis with the bark beetle showed that 25 % of the blast hits were unique to *R. ferrugineus*, indicating a higher, more complete transcript coverage for *R. ferrugineus*. We categorized the RPW ORs into seven subfamilies of coleopteran ORs and predicted two new subfamilies of ORs. The OR protein sequences were compared with those of the flour beetle, the cerambycid beetle and the bark beetle, and we identified coleopteran-specific, highly conserved ORs as well as unique ORs that are putatively involved in RPW aggregation pheromone detection. We identified 26 Minus-C OBPs and 8 Plus-C OBPs and grouped *R. ferrugineus* OBPs into different OBP-subfamilies according to phylogeny, which indicated significant species-specific expansion and divergence in *R. ferrugineus*. We also identified a diverse family of CSP proteins, as well as a coleopteran-specific CSP lineage that diverged from Diptera and Lepidoptera. We identified several extremely diverged IR orthologues as well as highly conserved insect IR co-receptor orthologous transcripts in *R. ferrugineus*. Notably, GR orthologous transcripts for CO_2_-sensing and sweet tastants were identified in *R. ferrugineus*, and we found a great diversity of GRs within the coleopteran family. With respect to SNMP-1 and SNMP-2 orthologous transcripts, one SNMP-1 orthologue was found to be strikingly highly expressed in the *R. ferrugineus* antennal transcriptome.

**Conclusion:**

Our study presents the first comprehensive catalogue of olfactory gene families involved in pheromone and general odorant detection in *R. ferrugineus*, which are potential novel targets for pest control strategies.

**Electronic supplementary material:**

The online version of this article (doi:10.1186/s12864-016-2362-6) contains supplementary material, which is available to authorized users.

## Background

Animals produce ‘chemical signature mixtures’ [1], released as volatile molecules, that are used to locate potential food resources, find mates, conduct social interactions and detect predators and pathogens [2]. Olfaction plays a key role in many aspects of animal behaviour including foraging, prey avoidance, locating oviposition sites, and mate recognition. Odorant-based chemical communication in insects has played a pivotal role in maintaining their status as the most species-rich group of animals on Earth [3]. A sophisticated olfactory system for detecting and interpreting odorants in the environment is a prerequisite for the survival and reproduction of insects [2, 4, 5]. The Coleopteran (beetles and weevils) and Lepidopteran (moths and butterflies) insect orders are the most species-rich animal orders on Earth [6]. In moths, chemical communication is a major factor in premating isolating, and a sex pheromone underlies reproductive behaviour and mating [7]. In beetles, olfaction plays a major role in finding mates and locating oviposition sites; however, it contributes even more to foraging for food resources, aggregation and prey avoidance [4]. In all of these insects, olfaction is well utilized at different levels, beginning with the reception of ligand at the periphery, the processing of signals at the antennal lobes, the merging of olfactory signals and other sensory modalities in the processing centres of the brain, and, finally, the rendition of olfactory signals into behaviour [4]. The economic importance of these insects can be attributed in part to the sensitivity and selectivity of their olfactory systems, which are important for locating their favoured hosts (plants and animals). The evolution of a highly sensitive and adaptable olfactory system is believed to be a key factor that has allowed insects to spread into virtually every environment on Earth [8].

Insects can sense bitter, sweet, and salty tastants, odours, pheromones, humidity, carbon dioxide, and carbonated water [9–12]. Insects detect odorants primarily in the antennae, and on the surface of the antennae there are thousands of olfactory sensilla (long trichodea, short trichodea, basiconica, coeloconica, and chaetica) that contain olfactory receptor neurons (ORNs) [8, 11]. Odorant molecules are detected by the antennae, and these events are transformed into electrical signals that are further processed by the central nervous system. The ORNs are the primary units of olfaction and can generally be divided into distinct classes based on their response spectra. Insect olfaction is initiated when volatile odours bind to odorant-binding proteins (OBPs) to cross the sensillum lymph surrounding the olfactory neuron dendrites. Because volatile odorants are highly hydrophobic, and because odorant-degrading enzymes (ODEs) are present in the sensillum lymph, odorants do not pass easily through the sensillum lymph surrounding the dendritic membranes of the ORNs. Hence, OBPs reversibly bind odorant molecules (ligands) in a protective cleft, carry them to the receptor protein and protect them from ODE breakdown [13]. A large number of soluble secreted proteins are also found in the sensillum lymph, including chemosensory proteins (CSPs), which are expressed over the entire body of the insect. Finally, these odorant carrier proteins deliver ligand molecules to the olfactory receptors (ORs) located in the dendritic membranes of receptor neurons [4]. Three types of insect chemosensory receptor proteins have been identified: odorant receptors (ORs), ionotropic receptors (IRs) and gustatory receptors (GRs). Insect ORs are heteromultimer formed by two proteins, an OR and a ubiquitous coreceptor (Orco). The binding of odorant molecules by the OR–Orco receptor complex opens the channel, and the chemical signal is then transformed into an electric signal that is transmitted to the brain [14–16]. IRs are three trans-membrane proteins homologous to the ionotropic glutamate receptor family ligand gated ion channels in vertebrates. IRs are narrowly tuned for amines and acids, whereas ORs are broadly tuned for esters and alcohols [17–19]. GRs are seven trans-membrane proteins that are distantly related to ORs and are expressed in the antennae, proboscis, palps and tarsae. They are tuned for CO_2_ detection and taste (bitter and sweet) [20]. Sensory neuron membrane proteins (SNMPs) are another class of proteins involved in pheromone reception at the ORNs; they are located in the dendritic membranes of pheromone sensitive neurons. SNMPs are thought to trigger ligand delivery to receptors [21]. SNMPs are members of the CD36 family of proteins and may function during the binding and transport of hydrophobic ligands [22].

Although substantial developments have been made in our knowledge of the molecular mechanism of odorant detection in insects, many previous studies were based on *Drosophila* and *Bombyx mori*, and information about the molecular basis of olfaction in coleopterans is sparse. There is still no consensus about the exact function of each olfactory gene family in Coleoptera, and very few studies have reported on the olfactory gene families in coleopteran insects [23–25]. Coleopteran insects are highly diverse and have adapted to live in a wide range of environmental conditions. Certain species cause major economic losses in many agricultural crops. Hence, the study of the olfactory senses that lead to their fitness is of the utmost importance for developing successful pest control strategies.

Palm trees are cultivated all over the world. The Red Palm Weevil (RPW) *Rhynchophorus ferrugineus* (Coleoptera: Curculionidae), the most harmful invasive insect species to palm trees, causes major economic losses in the cultivation of coconut, date and oil palms. The date palm is an extremely important fruit crop in Middle Eastern countries, where the RPW poses a serious threat, causing heavy losses every year. An infestation starts when an adult females lays eggs in the cuts, wounds, cracks and crevices in the trunk of the palm tree, from the collar region near the roots to the base of the frond petioles/axils near the crown. The RPW larvae bore into soft tissues such as the tree crown, the base of the petioles, the trunks of young palms or the rotting tissue of waning palm trees [26]. When RPWs attack a tree, individual insects are generally able to locate the tree with a male aggregation pheromone (composed of 4-methyl-5-nonanol and 4-methyl-5-nonanone), and this signal leads to a coordinated mass-attack that often leads to the death of the palm tree [27]. Recently, the United States Department of Agriculture (USDA) listed the RPW as a highly dangerous invasive species of significant quarantine importance [28]. Because of the ecological and economic effects of this pest, extensive knowledge regarding its olfactory communication should be collected. Investigations on the molecular mechanism of olfaction in the RPW provide insights into chemoreception in the RPW and may be useful for understanding odour detection in other coleopterans. In the present study, we used high-throughput sequencing to identify highly expressed transcripts of OBPs, CSPs, ORs, IRs, GRs and SNMPs in the RPW antennae, and our results will be fundamental for the functional characterization of the chemosensory proteins in RPW. Enhanced knowledge of the molecular mechanism of olfaction and the functional characterization of the receptor proteins in RPWs could ultimately lead to the identification of new targets for olfactory disruption and the development of safe pest control strategies.

## Methods

### Ethics statement

The red palm weevil collections were made with the direct permission of a cooperating land owner [Al-Kharj region (24.1500° N, 47.3000° E) of Saudi Arabia] and weevil culture was maintained in our laboratory as mentioned below. We reaffirm that none of the RPW collections were from National Parks or protected wilderness areas. Besides, these weevils are definitely not an endangered species.

### Insect rearing and antenna collection

The red palm weevil culture was maintained in our laboratory on sugarcane stems as described previously [29]. Briefly, the emerged adults were sexed, separated and placed individually in round 90 mm × 110 mm (diameter × height) plastic jars, which were covered with perforated caps. The adults were introduced into rearing jars with two freshly split sugarcane pieces for feeding and egg laying. Two weeks after pupation, the cocoons were harvested from the sugarcane stems and individually incubated in round 70 mm × 90 mm plastic jars with perforated screw caps and were checked daily for adult emergence. After a short period of approximately 3–6 min in a freezer (-10 °C) for immobilization, the delicate antennae were cut from the bases of the adult insects. The dissections were performed under a simple light microscope, and the collected antennae were saved for RNA extraction (in RNA later: Ambion).

### Total RNA extraction, cDNA library construction and Illumina sequencing

The antennae of ~60 *R. ferrugineus* 2 to 10-day-old adult beetles were excised, and total RNA was extracted from the antennae using a NORGEN purification kit (NORGEN Biotek Corp., Canada) according to the manufacturer’s instructions. The quantity and quality of the total RNA were validated using a Qubit® 2.0 Fluorometer (Invitrogen, Life Technologies), and the RNA integrity was further confirmed using a 2100 Bioanalyzer (Agilent Technologies). The aim of this study does not include a quantitative analysis of the chemosensory gene families in the *R. ferrugineus* antenna and therefore biological replicates were not considered.

Paired-end cDNA libraries were prepared using Illumina protocols and sequenced on the Illumina HiSeq platform. Briefly, a cDNA library was constructed using a TruSeq™ RNA Kit (Illumina Inc.), including purification of mRNA, fragmentation of total RNA, synthesis of the first and second strands of cDNA, cDNA end repair and adenylation at the 3′end, and adapter ligation and cDNA fragment enrichment. The products were purified and enriched by PCR to create the final cDNA library. Finally, the cDNA library was validated and quantified by a Qubit® 2.0 Fluorometer.

The HiSeq Illumina sequencing was performed at the core sequencing facility of the King Abdullah University of Science and Technology (KAUST), Jeddah, Saudi Arabia. The insert size of the library was approximately 306 bp. Image deconvolution and quality value calculations were performed using Illumina GAPipeline1.3.

### Data processing, assembly and annotation

A quality control step was first performed on the raw sequencing reads using the NGS QC Toolkit [30]. Standard RNA adapter sequences and regions of poor quality were clipped using the CLC Genomic Server and its ‘Trim Sequences’ tool. A *de novo* assembly was performed by the CLC Genomics Server using the ‘scaffolding’ and ‘mapping reads back to transcripts’ options. The resulting *de novo* assembled transcripts were locally searched against a non-redundant (*nr*) protein database using the BLASTx algorithm (e ≤ 0.001) in the standalone version of the BLAST+ tool [31], and results were stored in the BLAST archive format ASN.1. The results were subsequently translated into the required format (XML, tabular, pairwise) using the blast_formatter tool. The XML BLASTx results were imported into the Blast2GO annotation tool. Reads Per Kilobase per Million (RPKM) values were calculated for the assembled transcripts based on their mapping data according to the formula published in Mortazavi et al. [32].

### Gene identification and functional annotation

Following assembly, each transcript was identified by local or web-based searches using the NCBI BLASTx and BLASTn programs [33]. BLAST hits with e-values <1.0E − 5 were considered to be significant [34], and genes were putatively assigned to each contig based on the BLASTx hits with the highest score values. The BLAST XML files were uploaded to Blast2GO, and mapping, gene annotation, InterPro and KEGG analyses were performed [35, 36]. Each gene was functionally annotated with respect to its molecular function, biological process and cellular component.

The transcripts that contained errors leading to incorrect assembly were edited using Geneious v7.1.5 (http://www.geneious.com), and *de novo* assembly of the isotigs was performed. The open reading frame (ORF) of each unigene was determined by using the ORF finder tool (NCBI). InterPro analysis terms were assigned by Blast2GO [37] through a search of the *nr* databases. To annotate the pooled assembled transcriptome, we performed a BLASTx search against the *nr* NCBI, UniProtKB and KEGG databases using an e-value cut-off of 1.0E − 5.

### Comparative analysis of the beetle antenna transcriptome

The *Dendroctonus ponderosae* and *Ips typographus* (SRX132062 and ERX145718) [25] antennal transcriptomes were downloaded from NCBI, assembled with the CLC genomics server and saved as FASTA files. A comparative analysis of *R. ferrugineus*, *D. ponderosae* and *I. typographus* antennal transcripts was performed based on the results of best bidirectional BLASTx hit (reciprocal BLASTx, e-values <1.0E − 6). The objective of this study was to identify highly conserved and differentially expressed genes among the three coleopteran species.

### Candidate chemoreceptor proteins

BLASTx was used to search for ORs, Orcos, SNMPs, GRs and IRs in our NGS dataset. Candidate ORs, IR receptors and SNMP genes were identified by BLASTx and BLASTn searches. Sequence alignments were performed using the ClustalX program [38].

### Candidate genes involved in odorant transport

The putative odorant transport proteins OBPs and CSPs were identified by BLASTx and BLASTn searches as well as by searching for the “OBP sequence motif” C1-X_15–39_-C_2_-X_3_-C_3_-X_21–44_-C_4_-X_7_-_12_-C_5_-X_8_-C_6_ [39] and the “CSP sequence motif” C1-X_6–8_-C_2_-X_16–21_-C_3_-X_2_-C_4_ [40].

### Phylogenetic analysis of the candidate receptor proteins

The *R. ferrugineus* OBP, CSP, OR, SNMP, GR and IR nucleotide sequences were used as queries (BLASTx) to the GenBank database, and sequences from different insect species and their amino acids were retrieved and used to construct a phylogenetic tree. Similarity analyses of the DNA and protein sequences were conducted, and a multiple-sequence alignment was performed using the MUSCLE program [41], followed by manual inspection. A maximum likelihood analysis was performed, and a dendrogram was constructed using MEGA v6.0 [42]. The NCBI accession number for each gene is shown in the tree.

## Results and Discussion

### Illumina sequencing and *de novo* assembly

Illumina sequencing of the cDNA library prepared from the mRNA of the *R. ferrugineus* antennae produced a total of 194,157,678 raw reads with an average length of 101 base pairs (bp). Trimming adaptor sequences and eliminating low quality reads produced 187,637,735 reads (183,355,534 sequences in pairs and 4,282,200 single sequences) with an average length of 100 bp (Table 1). The raw reads were deposited at the National Center for Biotechnology Information (NCBI) Sequence Read Archive (SRA) database with the accession number SRX877682. After assembly, with scaffolding, there were 35,667 transcripts with an average length of 857 bp and a maximum length of 42,211 bp. The complete transcriptome size was 30.5 Mb, and the N50 size was 1557 bp, with 23 % of the sequences longer than 1 kb. The transcriptome Shotgun Assembly (TSA) project was deposited at DDBJ/EMBL/GenBank under the accession GDKA00000000. A comparison of the antennal transcriptome of *R. ferrugineus* with those of *D. ponderosae* and *I. typographus* [25] is presented in Table 1.

**Table 1:** Comparative summary of *R. ferrugineus, D. ponderosae* and *I. typographus* antennal transcriptome sequencing assembly and annotation

	*R. ferrugineus*	*D. ponderosae*	*I. typographus*
Raw reads	194,157,678	SRR449538: 75,425 SRR449539: 525,846 SRR449540: 524,467	ERR169822 :341,518 ERR169829: 4,111,071
		Total:1,125,738	Total: 4,452,589
Clean reads	183,355,534	SRR449538: 73,323 SRR449539: 504,320 SRR449540:504,853	ERR169822: 334,360 ERR169829: 3,084,804
		Total: 1,082,496	Total: 3,419,164
Singletons/ Unassembled reads	65,445,353	205,887	1,796,851
Number of contigs	35,667	15,396	21,014
Scaffolded metrics		1^a^	1^a^
-N50	1557 bp	1,521 bp	622 bp
-average	857 bp	1,070 bp	529 bp
-Max	42211 bp	8,088 bp	5,386 bp
-Min	101 bp	41 bp	31 bp

1^a^ Scaffolding not possible, only 454 or Illumina single reads used for assembly

### Functional annotation

The assembled transcripts were used as queries in BLASTx against the non-redundant (*nr*) NCBI protein database, UniProtKB, FlyBase and KEGG, with an e-value cut-off of 10E − 5. In general, the sequences had e-values between 1.0E − 4 and 1.0E − 10 (Additional file 1: Figure S1A). The sequence similarities between the *R. ferrugineus* antennal transcripts and the databases ranged from 32 to ~100 % (value: 1940) with a peak at 70 % (value: 7604) (Additional file 1: Figure S1B). A Blast2GO analysis of the 35,667 antennal transcripts of *R. ferrugineus* identified 2876 transcripts with blast hits, 18,338 without blast hits, 2713 with mapping results and 11,740 annotated sequences (Additional file 3: Figure S3A). The sequences without BLAST hits may have low similarity to functionally similar genes in the database, to novel genes or to parts of the 5′or 3′UTR regions or a large percentage of heavily spliced genes. The antennal transcripts of *R. ferrugineus* produced the most significant hits to the sequences of *Dendroctonus ponderosae*, followed by the sequences of *Tribolium castaneum* (Additional file 1: Figure S1C). The evidence code distribution for the BLAST hit chart indicates over-representation of the Inferred Electronic Annotation (IEA) code, followed by the Inferred by Mutant Phenotype (IMP) and Inferred by Direct Assays (IDAs) codes (Additional file 2: Figure S2A). The highest evidence code for the individual sequences was IEA, followed by IMP and, lastly, IDA (Additional file 2: Figure S2B). The majority of the functional predictions from the coding sequences were obtained from UniProtKB, followed by FlyBase (FB) (690,778 and 79,919 predictions, respectively) (Additional file 2: Figure S2C).

By using Blast2GO to search the *nr* database, GO terms were assigned, and an InterProScan search resulted in ~14,313 InterPro transcripts and 21,354 ‘without InterPro’ transcripts with an average length of 857 bp according to GO-annotation. Using this method, 7108 transcripts were assigned to one or more GO terms. ANNEX was run after BLAST, and the InterProScan results were annotated, resulting in the following: 66,040 total original annotations, 5256 new annotations, 908 original annotations replaced by new annotations due to specificity, and 2259 confirmed annotations.

A total of 3176 enzymes encoded in The KEGG returned cut-off BLAST hits > 1.0E − 5. A KEGG metabolic pathway analysis revealed 2687 transcripts that could generate 124 predicted pathways. The major enzyme commission (EC) classes that were covered include the oxidoreductases (709 transcripts), transferases (1053 transcripts), hydrolases (1187 transcripts), lyases (146 transcripts), isomerases (97 transcripts) and ligases (186 transcripts).

### GO analysis of the genes expressed in the *R. ferrugineus* antenna

The *R. ferrugineus* antennal transcriptome was GO-annotated based on matches to InterPro proteins. The annotation results and the distribution, GO-level distribution, number of GO-terms for the *R. ferrugineus* sequences with a specific length (x-axis), annotation score distribution and percentage of the *R. ferrugineus* sequences with a specific length (x-axis) are depicted in Additional file 3: Figure S3. The proteins with associated GO terms such as “molecular function”, “biological process” and “cellular component” were grouped and recorded at different match levels (Additional file 3: Figure S3C). The “cellular process” (6463) and “metabolic process” (5490) GO categories had the most abundant transcripts within the biological process GO ontology (Additional file 3: Figure S3C). In the “cellular components” GO category, the most abundant transcripts were involved in ‘binding’ (4797) and ‘catalytic activity’ (4375) (Additional file 4: Figure S4B). In the “cellular components” GO category, the transcripts were mainly associated with the terms ‘cell’ (5307) and ‘cell part’ (3993) (Additional file 3: Figure S3C). Of the proteins with *nr* database matches, ‘binding proteins’ were the most abundant protein class. Other highly abundant proteins included oxidoreductase proteins, kinases, peptidases, cytoskeletal proteins, ribosomal proteins and proteins involved in other major functional categories. Of the direct GO counts identified for the “biological process” ontology, ‘biosynthetic process’ and ‘cellular nitrogen compound metabolic process’ were among the first 20 dominant terms (Additional file 4: Figure S4A). Of the categories enriched for the direct GO counts identified as “cellular component”, ‘protein complex’, ‘nucleus’ and ‘cytoplasm’ were the most highly represented terms (Additional file 4: Figure S4B). In the “molecular function” ontology, 3607 transcripts with binding functions and 1037 transcripts with catalytic activities were annotated (Additional file 4: Figure S4C).

### Transcript abundance in the *R. ferrugineus* antenna

A summary of the highly expressed transcripts in the *R. ferrugineus* antenna is shown in Table 2. The highly expressed transcripts included apidermin (APD) protein, a cuticular protein family of insects, (Total read count: 2272597) [43] and OBP13 [25]. The OBPs (contigs 12, 23, 107, 226 and 382), and CSPs (contig 304) were highly expressed in the antenna with 8,614, 8,209, 4,111 3,548 and 3918 RPKM, respectively, indicating their roles in odorant reception (Table 2). Additional highly abundant transcripts were found on contig 140, encoding cytochrome P450 at 2684 RPKM, which had 63 % identity with *D. ponderosae* cytochrome P450 CYP345e2 (GenBank: AFI45008) [25]. Major house-keeping genes such as elongation factor, cytochrome c oxidase subunit I and cytochrome c oxidase I were highly expressed in the antenna of *R. ferrugineus* (Table 2). Interestingly, heat shock protein 70 was highly expressed in the *R. ferrugineus* antenna, and it showed 97 % similarity to *Lissorhoptrus oryzophilus* (AHE77377).

**Table 2:** The most abundant mRNA in *R. ferrugineus* antenna

Sequence name	Sequence description	Sequence length	Hit ACC	E-Value	RPKM	Bit-Score
RPW1_contig_4	apd-3-like protein	1338	ENN79298	1.29E-32	13900.2	133.65
RPW1_contig_18	hypothetical protein D910_08054	4349	ERL90707	3.82E-11	10336.8	77.411
RPW1_contig_12	13 kda hemolymph protein a	1293	AGI05170	2.63E-29	8614.5	120.553
RPW1_contig_23	pheromone binding protein	643	AAQ96921	4.23E-60	8209.6	195.282
RPW1_contig_186	saposin isoform 1	301	ESO85794	5.91E-10	7446.7	64.6994
RPW1_contig_107	odorant-binding protein 4	703	AAD31883	6.02E-62	7053.9	201.06
RPW1_contig_51	cg41536 cg41536- partial	8440	EFA11647	7.72E-84	6961.7	277.715
RPW1_contig_37	adp atp translocase	1215	ENN77548	0	4350.9	562.762
RPW1_contig_180	elongation factor 1 alpha	1733	NP_001107835	0	4123.9	905.975
RPW1_contig_226	13 kda hemolymph protein a	654	AGI05182	5.20E-36	4111.3	134.42
RPW1_contig_304	chemosensory protein 11	570	ENN78115	5.30E-63	3918.0	202.986
RPW1_contig_382	odorant-binding protein 28	652	AGI05178	1.70E-12	3548.1	71.2478
RPW1_contig_134	cytochrome c oxidase subunit i	2396	YP_006576018	3.68E-166	3334.9	332.028
RPW1_contig_331	hypothetical protein YQE_05135, partial	498	ENN78332	1.16E-11	3104.8	62.3882
RPW1_contig_140	cytochrome p450 6k1-like	1561	AFI45008	1.60E-149	2684.5	449.514
RPW1_contig_52	cral trio domain-containing protein	886	XP_971158	5.31E-24	2668.7	108.227

### Comparative analysis of the antennal transcripts

By comparing *R. ferrugineus* antennal transcripts with *D. ponderosae* and *I. typographus* transcripts in the SRA database, a large number of antennal transcriptome sequences were found to be identical. After assembly using the same parameters as used for *R. ferrugineus*, we obtained 15,396 and 21,014 unigenes from *D. ponderosae* and *I. typographus*, respectively (Table 1).

**Fig. 1:**
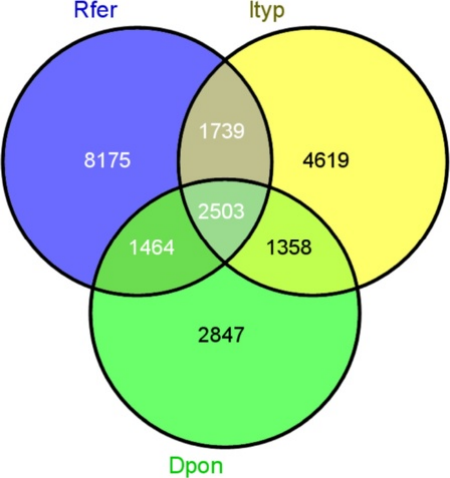
Venn diagram showing comparative analysis of *R. ferrugineus* antennal transcriptome. Comparative analysis of *R. ferrugineus* (Rfer) antennal transcriptome with those of *D. ponderosae* (Dpon) and *I. typographus* (Ityp) [25]. The diagram shows the blast homology genes in the antenna of *R. ferrugineus, D. ponderosae* and *I. typographus*. The comparative analyses antennal unigenes were performed based on the best bidirectional hits results (reciprocal BLASTx, e-value less than 1.0E − 6)

We selected the top bidirectional hits for each transcript from *R. ferrugineus*, *D. ponderosae* and *I. typographus* (producing a total of 13,881, 8,172 and 10,219 blast hit results, respectively) for the comparative analysis. When comparing the *R. ferrugineus* antennal transcripts with *D. ponderosae* and *I. typographus*, we found that 17 % of the top blast hits were shared between *R. ferrugineus* and the other two coleopterans, and 25 % of the blast hits were unique to *R. ferrugineus* (Fig. 1). A comparison of *R. ferrugineus* and *D. ponderosae* showed that 18 % of the blast hits were shared, and 45 % of the blast hits were unique to *R. ferrugineus* (Fig. 1). Similarly, a comparative analysis of the blast hits of *R. ferrugineus* and *I. typographus* showed that 18 % of the blast results were shared, and 44 % of the blast hits were unique to *R. ferrugineus. R. ferrugineus*, *D. ponderosae* and *I. typographus* shared 8 % of their blast hits, whereas *R. ferrugineus* and *D. ponderosae* shared 6.6 % of their blast hits, and *R. ferrugineus* and *I. typographus* shared 7.2 % of their blast hits (Fig. 1). In total, we identified 2,993 highly conserved genes between *R. ferrugineus, D. ponderosae* and *I. typographus*. Of these genes, 491 were duplicated, or were predicted to be splice variants or partial sequences (Additional file 5: Table S1). Because the RPW genome is not available, alternative splicing was not predicted. However, multiple alignment of *R. ferrugineus* conserved genes produced the same BLASTx results. Interestingly, we found splice variants in a few cases (Additional file 5: Table S1). One of the candidates among the conserved genes is a group of several orthologous sequences of Heat-shock proteins (HSP), including the heterogeneous HSP family of small heat shock proteins (sHSP) (Additional file 5: Table S1), which play an important role in protecting organisms from stress [44]. Diversification and evolution of HSP is of critical importance in increasing cold/heat stress resistance and has implications for adapting to climate change [45, 46]. The chemosensory candidates among the conserved gene orthologous are OR- 2, 10 and 23; OBP-30; CSP-10 and 19; SNMP-1; GR-54 and 64 and several IR candidate genes (Additional file 5: Table S1). A large portion of the *R. ferrugineus* transcripts (25 %) had no hits in the available antennal transcriptomes of the other two species. This may be due to the larger data set (35,667 transcripts) of *R. ferrugineus* and the lower coverage in the other studies (Fig. 1). Another possibility may be alternatively spliced genes. We were unable to remove the splice variants because the reference genome of *R. ferrugineus* was not available. The lack of blast hits for many *R. ferrugineus* transcripts in *D. ponderosae* or *I. typographus* may be due to novel genes with unique functions or highly conserved genes.

### Odorant binding proteins in *R. ferrugineus*

In the *R. ferrugineus* antennal transcriptome, a total of 38 OBP transcripts were identified based on BLASTx results and the “OBP sequence motif” C1-X_25–68_-C2-X3-C3-X_31–46_-C4-X_8–29_-C5-X_8_-C6 (where X is any amino acid) [47–50] (Additional file 6: Figure S5). A six-cysteine protein structure is the most characteristic feature of insect OBPs [39]. The arrangement pattern of the conserved cysteines in the *R. ferrugineus* OBP family is comparable to the patterns in other insect OBPs [50]. An RPKM value analysis revealed that seven OBPs (RPW_Unigene_1, contigs_23, 107, 226, 382, 257 and 446) are highly abundant transcripts in the *R. ferrugineus* antennal transcriptome (RPKM >1000) (Additional file 7: Figure S6A). We followed the previously proposed OBP naming system [51], which refers to OBPs missing C2 and C5 as Minus-C OBPs, and OBPs carrying more than six conserved cysteine residues as Plus-C OBPs. In the *R. ferrugineus* antennal transcriptome data, we identified twenty six Minus-C OBPs that are missing the second and fifth cysteines (Additional file 6: Figure S5), eight Plus-C OBP members, including eight OBPs carrying additional conserved cysteines located upstream of C1 (RPW_unigene_1, contigs_382, 3997, 446, 14025, 14511, 19755 and 257), and two OBPs carrying conserved cysteines between C1 and C2 (contig_23691 and contig_14511). An alignment of the *R. ferrugineus* OBP proteins shows low average pairwise sequence identity between the OBP family members (Additional file 6: Figure S5). The arrangement pattern of the conserved cysteines (C1-C6) of the contigs 23, 23127, 107, 446, 4661, 23691, 33721, 14511, 16551, 29, 3199 and 3213 of C1 to C6 are similar to the spacing patterns when all six cysteines are present (Additional file 6: Figure S5).

**Fig. 2:**
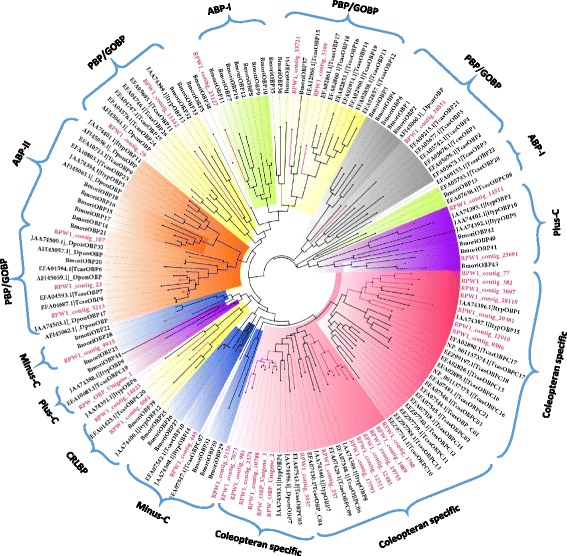
Maximum likelihood (ML) tree of the odorant binding protein (OBP). *Bombyx mori* OBPs [50] were used as reference to classify the *R. ferrugineus* OBPs and the ML analysis was computed using MEGA (v.6.0) [42] (JTT model for ML heuristic searches methods was Nearest-Neighbor-Interchange). The coleopteran OBPs; *T. castaneum* OBPs, *D. ponderosae* and *I. typographus* [25] were also used to construct the tree with *R. ferrugineus* OBPs. *R. ferrugineus* OBPs transcripts and node are marked with red color. *R. ferrugineus* OBPs putatively identified are highlighted. GenBank accession numbers are indicated. Scale 0.4 amino acid substitution per site

A phylogenetic tree based on neighbour-joining (NJ) is shown for the *R. ferrugineus* OBPs in Additional file 8: Figure S7. A phylogenetic tree was also constructed using the maximum-likelihood method, and we identified six possible insect OBP subfamilies by following the a previously described method [51]; in addition, *R. ferrugineus* OBPs were putatively identified based on comparison with *B. mori* OBPs [50] (Fig. 2). Based on phylogeny, one candidate ABPI (contig_23127), two candidate Plus-C (contig_14511 & contig_23691), four candidate CRLBPs (contigs_33721, 4661, 14025 and 3199), four candidate ABP II (contigs_107, 29, 23 & 3213) and three candidate Minus-C (contigs_9915, 446 & 11442) were identified (Additional file 8: Figure S7). The phylogenetic tree shows that RPW1_contig_16551 is closely related to RPW1_contig_29 (ABPII subfamily) and possesses insect PBB-GOBP protein domains; hence, it is included within the ABPII subfamily (Additional file 8: Figure S7). Based on the BLASTx analysis, contig_23127, contig_107, contig_29, contig_23 and contig_3213 (Fig. 2) contain insect pheromone/odorant binding protein domains and are placed in the PBP_GOBP family (pfam01395); however, they share only 16 % identity. RPW1_contig 23 and RPW1_contig 3213 show a 54 % amino acid identity with the Scarab beetle *Anomala octiescostata* (BAC06497) and *A. cuprea* (BAC06496), respectively, which are involved in the detection of sex pheromone compounds [52]. RPW1_contig_23127 is in the ABP I family [51], and shows a 35 % identity with OBP5 of *Helicoverpa armigera*, and it has been reported that HarmOBP5 is expressed in the antenna and leg of *H. armigera* [53]. The OBP family includes the pheromone-binding proteins (PBPs) and the general odorant-binding proteins (GOBPs); the former are responsible for transporting pheromone molecules, and the latter transport general odorants such as plant volatiles. The PBPs in dipterans and lepidopterans have been characterized, but little characterization has been conducted for the PBPs in coleopterans [4].

We observed sound bootstrap support in the ABPII subfamilies and slight bootstrap support in the other subfamilies of the OBPs in *R. ferrugineus* (data not shown). The five ABPII OBPs share an average of 31 % identity. The CRLBP subfamily represents four monophyletic OBPs and includes *B. mori* OBPs (BmoriOBP34-37 and BmoriOBP39), *T. castaneum* OBPs (TcasOBP12-20) and *I. typographus* OBPs (ItypOBP6 and 12). The four CRLBP *R. ferrugineus* OBPs share an average of 23 % identity. As reported earlier in *D. melanogaster* [51], the Minus-C subfamily of the *R. ferrugineus* OBPs represents a significant number of monophyletic (except contigs 11442 and 14025) antennal transcripts (26 transcripts total) that all lack a conserved C2 and C5 and are predicted to be pseudogenes, non-olfactory OBP-like proteins, or novel OBPs that diverged from moth OBPs and are unique to *R. ferrugineus* (or coleopterans). These proteins need to be characterized. The 26 Minus-C OBPs share an average of 33 % identity. Based on phylogeny, we identified contig_23691 and contig_14511 as part of the Plus-C subfamily, and we found that contig_23691 carries two additional cysteines between C1 and C2, and contig_14511 carries additional cysteines upstream of C1 and downstream of C6. Based on a multiple sequence alignment, we identified eight Plus-C subfamily members (RPW_unigene_3, contigs_382, 446, 3997, 14025, 14511, 19755 and 257) whose sequences share an average of 47 % identity. All of the Plus-C subfamily members exhibit six conserved cysteine residues, C1-C6, and they also carry an additional conserved cysteine located upstream of C1 or downstream of C6 (Additional file 6: Figure S5).

We built an ML tree based on an alignment of the OBP sequences from six species: *R. ferrugineus, B. mori, D. melanogaster, D. ponderosae, T. castaneum* and *I. typographus.* These species represent three orders (Fig. 2). The six subfamilies for *R. ferrugineus* defined above were also found in the clades with *B. mori* OBPs (Fig. 2). The highly abundant OBP transcript RPW_unigene_1 forms a cluster with TcasOBPC19 (EFA10683) [54]. The other coleopteran-specific OBPs that form clades with *T. castaneum, D. ponderosae* and *I. typographus* include RPW1_contig_14025 (TcasOBPC19), RPW1_contig_3213 (DponOBP17), RPW1_contig_23 (TcasOBP7), RPW1_contig_29 (DponOBP: AFI45061), RPW1_contig_11442 (ItypOBP13), RPW1_contig_3199 (TcasOBP12-17), RPW1_contig_16551 (TcasOBP21, 1–5), RPW1_contig_14511 (ItypOBP2), RPW1_contig_28119 (ItypOBP1), RPW1_contig_29381 (ItypOBP15), RPW1_contig_257 (ItypOBPC7), RPW1_contig_3937 (TcasOBPC05) and RPW1_contig_446 (ItypOBP14). The number of *R. ferrugineus* OBPs detected in the present study is much higher than in two other curculionids, *D. ponderosae* and *I. typographus* (with 31 and 15 OBPs, respectively) [25]. The phylogenetic comparison also showed that several of the unique OBPs in *R. ferrugineus* antennae form two monophyletic groups (Fig. 2). The first group includes RPW_unigene_2 and 3, contigs 1768, 1689, 19755, 12481, 12511, 17793, 10788, 2374, 981, 257, 3937, 7073 and 9136, which form a cluster with TcasOBPC04, TcasOBPC05, TcasOBPC06, TcasOBPC09, DponOBP7, ItypOBP4, ItypOBP7 and ItypOBP8 (Fig. 2). The second group includes RPW1_contigs 77, 382, 3997, 28119, 29381, 12010 and 8586, which form a clade with ItypOBP15 and TcasOBP15, 16, 17 and 18 (Fig. 2). The *B. mori* OBPs are not represented in this clade. This result suggests that there was a lineage specific expansion of the OBPs in coleopterans and lepidopterans. This result also suggests that significant species-specific expansion and divergence have occurred in *R. ferrugineus*. In total, 38 putative OBPs were identified in *R. ferrugineus,* and their rich diversity indicates that *R. ferrugineus* OBPs play a fundamental role in odour detection and olfactory sensation. The genus *Rhynchophorus* is the foremost pest of palm trees in the world. The fact that adult weevils are able to locate food resources using plant volatiles as well as the range of volatiles (including aggregation pheromones) exploited by this insect are direct indications of the divergence of its OBPs and the sophistication of its chemical communication systems.

### Chemosensory proteins in *R. ferrugineus*

**Fig. 3:**
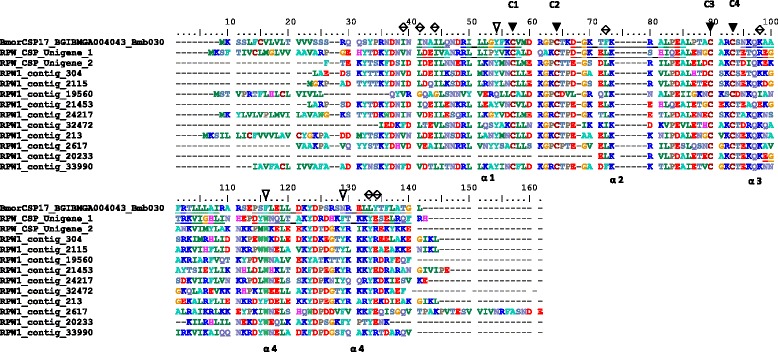
Alignment of the *R. ferrugenieus* CSPs contigs with *B. mori* CSP17. Highly conserved cysteine are shown with dark arrowhead above. Conserved three aromatic residues (54Y (Tyr), 116 W (Trp) or F (Phe) and 129Y or F) shown in hollow arrowheads. Hydrophobic residues at the two mouths of the channels are marked with double arrowheads, as reported with *B. mori* [58]. Alpha helical domains (1α-6α) in *R. ferrugineus* is identified based on the *B. mori*, and the amino acids underlined

Chemosensory proteins (CSPs) are approximately 100–200 residues long and present a conserved pattern of four cysteines forming two independent loops [55]. In this study, we identified 12 CSPs in *R. ferrugineus* antennae. Based on RPKM values, contig_304 is the most highly expressed of these antennal transcripts (RPKM > 3900); it shows high identity (76 %) with DponCSP8 in *D. ponderosae* (AGI05164) (Additional file 7: Figure S6B). Orthologous CSPs were also found in *T. castaneum* (TcasCSP7, 11 and 10). All of the *R. ferrugineus* CSPs have four highly conserved cysteine residues with a common spacing pattern (Cys-X_6_-Cys-X_18_-Cys-*X*_2_-Cys, where X represents any amino acid) (Fig. 3). The deduced CSPs share 20–63 % protein identity, showing that they belong to a diverse family of CSP proteins (data not shown). The protein sequence alignment shows that three aromatic residues (54Y (Tyr), 116 W (Trp) or F (Phe) and 129Y or F) are highly conserved within the *R. ferrugineus* CSP protein family (Fig. 3). These residues are most likely located at the binding site. It has been reported that these three aromatic residues block tunnel opening and closing and are present in all insect CSPs [56]. *R. ferrugineus* CSPs are conserved at Y 54, except for RPW_contig_19560, which was replaced by a non-aromatic residue, Gln (Q). All of the other CSPs have conserved aromatic residues at 116 and 129 (Fig. 3). The residue positions at 37 (D/Q), 61 (R/K), 67 (D/E), 73 (K), 83 (P/Q), 84 (E/D), 86 (L/I), 122 (K/Q) and 124 (D/A) are conserved. It has been reported that these functionally conserved residues are predicted to be involved in ligand binding in insect CSPs [57, 58]. The *R. ferrugineus* CSPs have diverged; however, they share moderate sequence identity and retain all of the conserved aromatic residues, hydrophobic residues and alpha helical domains of lepidopteran CSPs (Fig. 3).

**Fig. 4:**
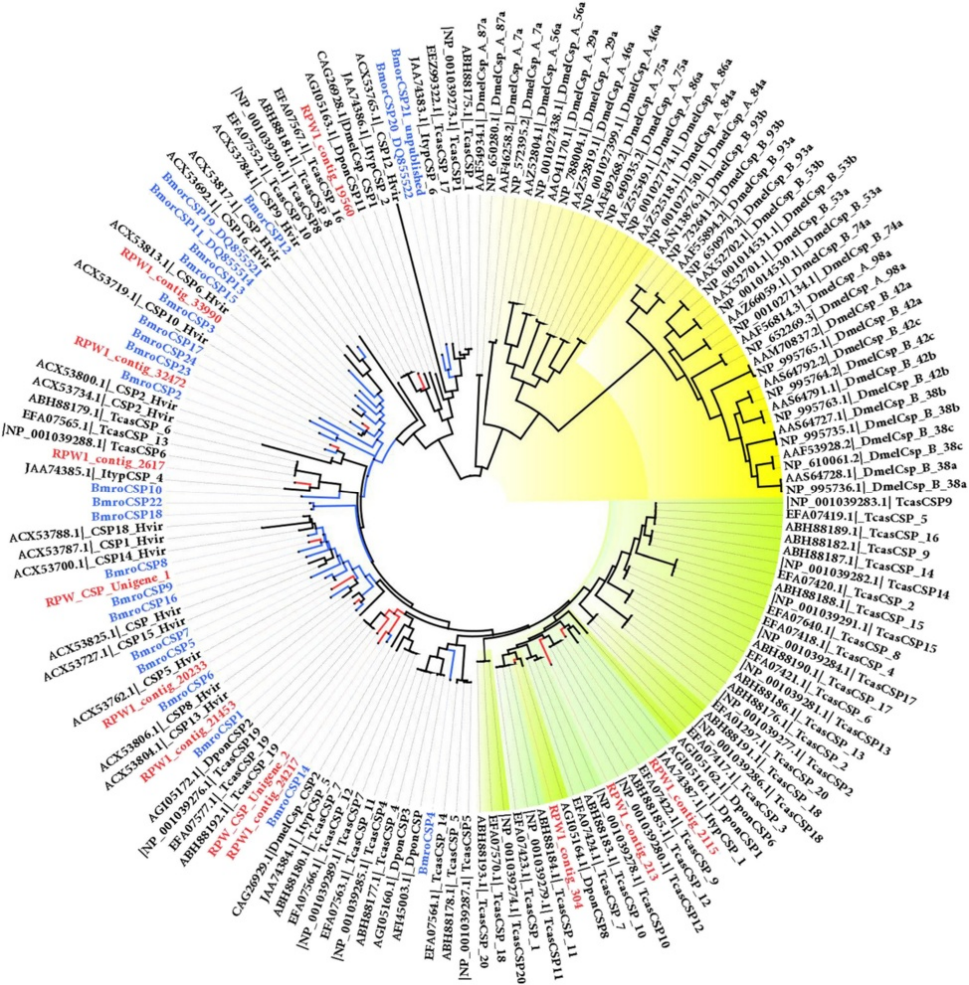
Maximum likelihood (ML) tree of the chemosensory proteins (CSP). *Bombyx mori* CSPs [58] were used as reference to identify the *R. ferrugineus* CSPs and the ML analysis was computed using MEGA (v.6.0) [42] (JTT model for ML heuristic searches methods was Nearest-Neighbor-Interchange). Insect CSPs: *D. melanogaster, H. virescens, T. castaneum*, *D. ponderosae* and *I. typographus* were also used for the construction tree. *R. ferrugineus* CSP transcripts and nodes are marked with red color and *B. mori* CSPs and nodes are with blue color. Coleopteran specific CSP clades are highlighted with yellow color. Dipteran specific CSP clade are highlighted with green. GeneBank accession nos are indicated. Scale 0.6 amino acids substitution per site

We built an ML tree based on an alignment of the CSP sequences in seven species, *R. ferrugineus, B. mori, H. virescens*, *T. castaneum, D. melanogaster, D. ponderosae* and *I. typographus*, which represent two orders (Fig. 4). Phylogenetic analysis identified *R. ferrugineus* CSPs that were orthologous to *B. mori* CSP14 (RPW_contig_24217), CSP1 (RPW_contig_21453), CSP6 (RPW_contig _20233), CSP9 (RPW_unigene_1), CSP2 (RPW_contig_32472) and CSP3 (RPW_contig_33990), clearly indicating that these genes evolved from a common ancestral insect gene and that they retain conserved residues in both orders (Fig. 4). CSPs are more highly conserved than OBPs across insect species and are widely expressed in different parts of the insect body [57, 58]. Surprisingly, the CSP clade representing *R. ferrugineus* contigs 20233, 32472, 33990 and RPW_CSP_unigene 1 forms a cluster with the *B. mori* and *H. virescens* CSPs without any CSP representatives from *T. castaneum, D. ponderosae* or *I. typographus* (Fig. 4). The *R. ferrugineus* CSP RPW_contigs 2115, 213 and 304 show high identity with DponCSP1, TcasCSP12 and DponCSP8, respectively, which form a separate clade that diverged from dipterans and lepidopterans. Hence, we assume them to be a coleopteran specific CSP lineage (Fig. 4). These CSPs may have important roles in *R. ferrugineus* physiology and semiochemical perception that need to be elucidated. Similarly, phylogenetic analysis also identified a dipteran-specific clade that did not include any of the CSPs in the coleopterans and lepidopterans, indicating an order-specific expansion of the CSP lineage (Fig. 4). Interestingly, *R. ferrugineus* CSPs (contigs 24217, 21453, 32472, 33990, 20233 and RPW_CSP_unigene 1) are more similar to the CSPs in *B. mori* and *H. virescens* than those in coleopterans (Fig. 4). This may be due to a lack of available information on coleopteran antennal CSPs [25, 54]. The number of *R. ferrugineus* CSPs identified in the current study is higher than the number of CSPs that have been identified in other two Curculionids, *D. ponderosae* and *I. typographus* (which have 11 and 6 CSPs, respectively) [25]. Undoubtedly, the CSPs that affect chemoreception in *R. ferrugineus* could have important roles as carriers in odorant perception. Further studies are needed to understand the potential roles of antennal CSPs in *R. ferrugineus* communication.

### Odorant receptors in *R. ferrugineus*

Volatile ligands are first captured by insect antennae. They are then absorbed through pores on the surface of the sensilla. Odorant receptors (ORs) expressed on the dendritic membranes of ORNs are activated by odorants alone or by odorant–OBPs complexes [4, 8]. Although ORs have been studied more intensively than other olfactory proteins, a mechanism for the binding of odorant ligands to odorant receptors has recently emerged in which ligand specific ORs require dimerization with the highly conserved gene OR83b or the Odorant coreceptor (Orco). In principle, odorant molecules bound by the OR–Orco receptor complex initiate channel opening. The Orco protein belongs to a seven transmembrane protein family that represents the only conserved OR protein among divergent insect species and is particularly expressed only in tissues harbouring olfactory sensory neurons [59].

**Fig. 5:**
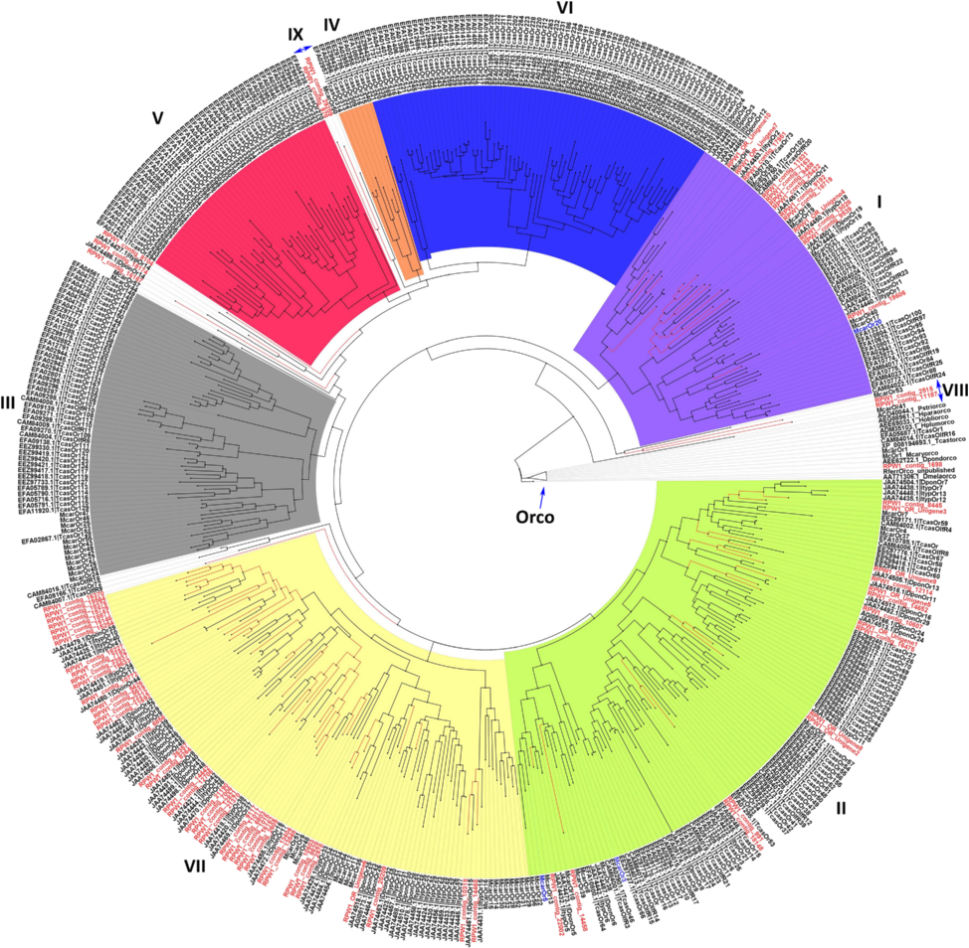
Maximum likelihood tree of candidate odorant receptor (OR) proteins from *R. ferrugineus*. ML analysis was computed using MEGA (v.6.0) [42] (JTT model for ML heuristic searches methods was Nearest-Neighbor-Interchange). Insect ORs: *T. castaneum*, *M. caryae, D. ponderosae* and *I. typographus* were also used for the construction of the tree. *R. ferrugineus* ORs transcripts and nodes are marked with red color. The branch containing *D. melanogaster* Orco was used as outgroup to root the tree. The different subfamily (numbered 1–7 according to Engsontia et al., [23]; Mitchell et al., [24]; Andersson et al., [25] colored group as indicated. Two new subfamilies (named VIII and IX) proposed as shown in the figure. GenBank accession nos are indicated. Scale 0.6 amino acid substitutions per site. Refer Additional file 10: Table S3 for the details of GenBank accession nos

Seventy-six ORs were annotated in the RPW antennae transcriptome, which is more than the number of ORs found in *D. ponderosae* (41 ORs) in the same Curculionidae family [25]. The average sequence length of the RPW ORs was 1,008 bp. Forty four of the ORs likely represented full length OR genes because they were longer than 1 kb in size, whereas 19 contig sequences were most likely partial sequences with sizes of less than 0.5 kb. The expression of the RPW ORs in the antenna transcriptome was relatively low compared with other contigs, as indicated by the average RPKM value of 4.97, whereas the highest RPKM was 235 (RPW contig 1698) and was observed in seven subfamilies (Fig. 5) (Additional file 7: Figure S6C). We built a phylogenetic tree to visualize the lineage-specific subfamily expansion of the RPW ORs from the highly conserved odorant coreceptor, DmelOrco/DmelOr83b (as a root) [23]. The tree was built using a total of 455 amino acid sequences from *T. castaneum* (52 %) [23], followed by RPW (16 %), *M. caryae* (12 %) [24], *I. typographus* and *D. ponderosae* (9 %) [25]. The lineage subfamily groupings agreed with previous studies of the above coleopterans.

The expressed RPW ORs were largely grouped into the three subfamilies 1, 2 and 7, which is similar to the groupings reported for *I. typographus* and *D. ponderosae* [25] (Fig. 5). Subfamilies 4, 5 and 6 exclusively consisted of *T. castaneum* ORs, whereas subfamily 3 exclusively contained *T. castaneum* and *M. caryae* ORs. We found that some of our RPW contigs were not grouped into the previously defined seven subfamily clade. Thus, we propose to classify them into two new subfamilies. Subfamily 8 contained two 2 RPW contigs (11187 and 2515) and one McarOR53. This subfamily serves as a root for all of the previously defined subfamilies (1–7) and has the closest lineage to the Orco subfamilies. Subfamily 9, consisting of RPW contigs 29259 and 978 together with McarOR50, was positioned on the clade of subfamilies 4 and 6. In addition to the two newly proposed subfamilies, we found that the RPW contigs 8104, 18174 and 16167 presented a different clade that expanded into three previously defined subfamilies (4, 5 and 6), in a similar manner to RPW contig 16370, which expanded to subfamilies 2 and 7.

Subfamilies 1 and 2 have a similar composition, and all of the coleopteran ORs were located in these clades. *T. castaneum* ORs are the majority OR in these clades (36.7 and 55.77 %, respectively); followed by RPW contigs (26.5 . and 14.42 %, respectively). Subfamily 7 is interesting because it is a novel subfamily proposed by Mitchell et al. [24], and it contains the majority of coleopteran ORs (except for *T. castaneum)*. In this seventh subfamily, the highest number of ORs were associated with RPW contigs (39 ORs), followed by *D. ponderosae*, *I. typographus* and *M. caryae* with 28, 25 and 16 ORs, respectively.

We currently have genome-scale information about the ORs in *Drosophila, Anopheles, Nasonia, Tribolium, Bombyx* and *Apis*. Genome analysis of the *D. melanogaster* revealed the presence of 62 olfactory receptors (ORs), which are encoded by 60 genes, and 68 gustatory receptors (GRs), which are also encoded by 60 genes [60–63]. In the *A. gambiae* genome, 79 olfactory receptors were previously identified [64]. The *A. mellifera* genome encodes 170 OR genes [65] and the *B. mori* genome encodes 41 olfactory receptors, 17 of which appear to be orthologs of *Helicoverpa virescens* [66–68]. The honey bee odorant receptor for the queen bee has been previously identified [65]. The *A. aegypti* genome was found to contain genes for 131 OR receptors [69]. A putative chemoreceptor family consisting of 26 ORs was identified in *T. castaneum* [23, 54, 70]. Recently, a characterization of the odorant receptors of the cerambycid beetle has been reported [24], including 57 putative ORs. In the present study, we have presented 76 antennal ORs in *R. ferrugineus* (the reference genome of *R. ferrugineus* was not available for the splice variant analysis, hence the actual OR number in *R. ferrugineus* may vary), and we have compared them with other OR protein families to determine that there are coleopteran-specific ORs as well as highly specialized *R. ferrugineus* ORs. The specialization of certain ORs signifies possible adaptation of *R. ferrugineus* to specialized ecological niches. Further functional characterization studies of *R. ferrugineus* ORs in a heterologous expression system are in progress.

### Pheromone receptors in *R. ferrugineus*

**Fig. 6:**
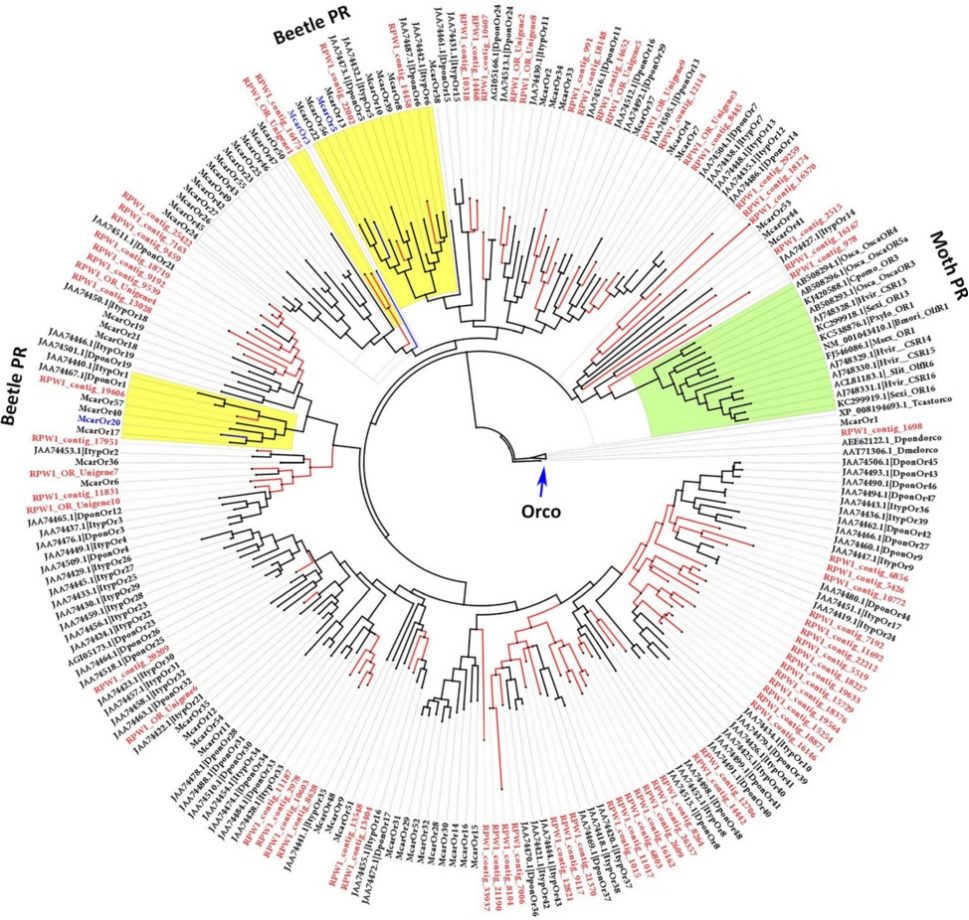
Maximum likelihood tree of candidate odorant receptor (OR) proteins from *R. ferrugineus* highlighting sex pheromone receptors and aggregation pheromone receptors. ML analysis was computed using MEGA (v.6.0) [42] (JTT model for ML heuristic searches methods was Nearest-Neighbor-Interchange). Insect ORs: *T. castaneum*, *M. caryae, D. ponderosae* and *I. typographus* were used for the construction of the tree. Functionally characterized moth pheromone receptors (*B. mori, O. scapulalis, M. sexta, S. exigua, Cydia pomonella, P. xylostella* and *H. virescens*) were included in the tree construction and PR clade is highlighted. *R. ferrugineus* ORs transcripts and nodes are marked with red color. *M. caryae* aggregation pheromone receptors and nodes are marked with blue color. The branch containing *D. melanogaster* Orco was used as outgroup to root the tree. The *M. caryae* aggregation PR [24] is highlighted with yellow and moth PR with green color. GenBank accession nos are indicated. Scale 0.5 amino acid substitutions per site

Pheromone molecules are perceived at the periphery of the olfactory system by pheromone-sensitive sensilla primarily located on the antennae [5]. Olfactory sensilla typically consist of three olfactory neurons with different diameters housed within a hair-like structure called the trichoid sensillum [71]. Pheromone molecules transported to the dendritic membranes of ORNs are recognized by pheromone receptors (PRs), which are a subclass of insect ORs [5]. In the present study, we found RPW aggregation pheromone candidate genes in the same clade as McarOR20 (sensitive to (2S,3R)-2,3-hexanediol) in RPW contig 19606, which is in subfamily 1, and those in the same clade as McarOR3 and McarOR5 (sensitive to (S)-2-methyl-1-butanol and 2-phenylethanol, respectively) [24] in RPW contigs 14458 and 22002, respectively (Fig. 6). McarOr20 shares 24 % amino acid identity with RPW1_contig_19606, McarOr3 shares 18.28 and 17 % identity with RPW1_contig_16475 and RPW_OR_unigene1, respectively, and McarOr5 shares 38 and 13 % identity with RPW1_contig_22002 and RPW1_contig_14458, respectively. A phylogenetic tree of coleopteran ORs (except *T. castaneum*) with identified sex pheromones (from moths) was built using DmelOrco as a root. Insect sex pheromone PRs were found to be grouped into one unique clade, which has the closest lineage to Orco clades (Fig. 6). Within this sex pheromone clade, there are 6 RPW ORs, RPW contigs 978, 16167, 2515, 16370 and 18174, that are potential pheromone receptor candidate genes (see Fig. 6). These six RPW contigs share 59–72 % amino acid identity with each other but share less than 20 % amino acid identity with moth PRs. The PR clade also includes McarOr41, McarOr44, McarOr53 [24] and ItypOr14 [25], as well as beetle PRs sharing 5–40 % amino acid identity. These results indicate that coleopteran PR proteins are highly diverged from moth PRs. The richness of the diversity of the beetle PRs indicates that they play a fundamental role in olfactory sensation related to pheromone detection. Although there have been no reports of sex pheromone compounds in *R. ferrugineus*, in light of our new results, an evaluation of the female sex pheromones (or any volatiles released during courtship) of the RPW using sequential SPME-GC/MS (solid phase micro extraction-gas chromatography mass spectrometry) is highly recommended.

In the silkworm, *B. mori* sex pheromone signals mediated by a specific combination of olfactory receptors are exclusively expressed in a pair of adjacent pheromone-sensitive neurons of the male antennae [72]. The candidate pheromone receptor of *B. mori* has been functionally characterized [73]. The functional characterization and identification of putative receptors for the main sex-pheromone components of the moth species *Plutella xylostella*, *Mythimna separata* and *Diaphania indica* [74] and *Manduca sexta* [75] have been reported. In the genus *Ostrinia* (Lepidoptera: Crambidae), pheromone receptors have been characterized primarily in *O. scapularis* and *O. nubilalis*, and receptors from different species have been functionally expressed in *Xenopus* oocytes [76–78]. An EST data collection from the male antennae of *Spodoptera littoralis* and *Heliothis virescens* identified several putative pheromone receptors [79, 80]. Studies on pheromone receptors are extremely important because any changes (mutations) in pheromone receptors may ultimately contribute to speciation events [81]. Although there are have been more than 10 pheromone receptors reported, there have been none from coleopterans, with the exception of Mitchell et al.*,* [24] who identified and functionally characterized the ORs in the cerambycid beetle *M. caryae* and found that they are tuned to three molecules: the receptor McOR3 is sensitive to (S)-2-methyl-1-butanol, McOR20 is sensitive to (2S,3R)-2,3-hexanediol, and McOR5 is sensitive to 2-phenylethanol. We predict that RPW contig 19606, which is an orthologous OR to Mcar20 of *M. caryae* [24] and may have a role in aggregation pheromone (4-methyl-5-nonanol and 4-methyl-5-nonanone) detection in *R. ferrugineus*. Further research on *R. ferrugineus* ORs, with a specific emphasis on molecular mechanisms, will facilitate better understanding of the behaviour of the RPW and will lead to a more informed approach to the management of this quarantine pest that inflicts significant damage every year on palm trees throughout the world.

### Ionotropic receptors in *R. ferrugineus*

Insect ionotropic glutamate receptors (IGluRs), a conserved family of synaptic ligand-gated ion channels involved in chemosensation, have recently been characterized in *D. melanogaster* [17]. The iGluR-family constitutes a distinct and divergent subfamily of ionotropic receptors (IRs) that are specifically expressed in insect antennae and function as chemoreceptors for the detection of a variety of chemical molecules [17, 82]. Another class of novel receptors that do not belong to the iGluR-family includes the α-amino-3-hydroxy-5-methyl-4-isoxazolepropionic acid (AMPA), kainate and N-methyl-D-aspartate (NMDA) receptors, which have divergent ligand-binding domains that lack the characteristic glutamate interacting residues [17]. IRs act in combinations of up to three subunits of individual odour-specific receptors and one or two of the broadly expressed coreceptors IR25a, IR8a, and IR76b [83]. The iGluR-subfamily has an important role in synaptic transmission because the proteins are receptors for the excitatory neurotransmitter glutamate; they have been well studied in vertebrates [84]. IRs have been characterized in *D. melanogaster* [17, 82], *B. mori* [82] and *Spodoptera littoralis* [85], but no such studies have been reported for coleopterans [19].

**Fig. 7:**
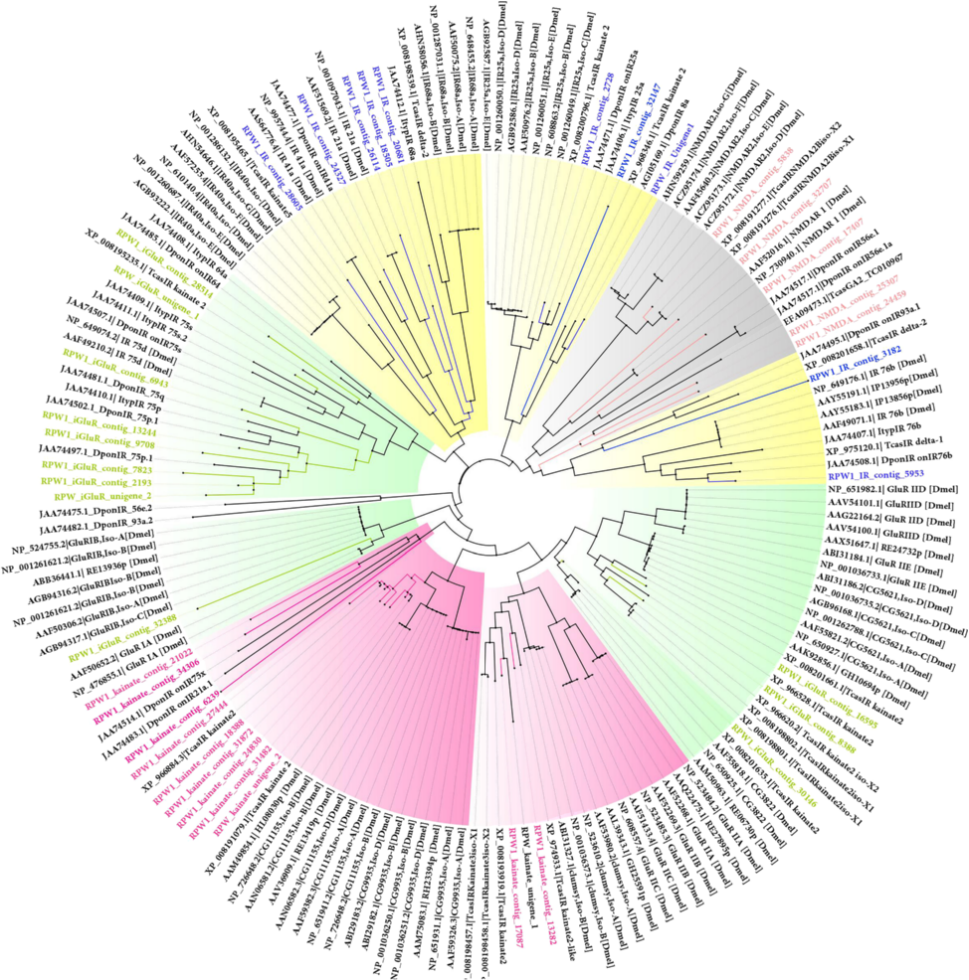
Unrooted tree of putative *R. ferrugineus* IRs with those of *D. melanogaster, T. castaneum*, *D. ponderosae* and *I. typographus* IRs. ML analysis was computed using MEGA (v6.0) [42] (JTT model for ML heuristic searches methods was Nearest-Neighbor-Interchange). *R. ferrugineus* candidate IR, iGluRs, kainite and NMDA depicted in blue, green, red and pink font. GenBank accession numbers are indicated. Each color represents a group of orthologous sequences and group name and node are colored. IRs, iGluRs, kainite and NMDA clades are highlighted with yellow, green, pink and grey colors, respectively. Scale bar represents the 0.4 amino acids substitutions per site

We identified 10 candidate IRs in the *R. ferrugineus* antennal transcriptome with very low expression levels in the *R. ferrugineus* antenna. However, RPW_contig_2728 was relatively highly expressed (RPKM: 48) (Additional file 7: Figure S6D). In the MSA and phylogenetic analyses, the identified IRs clustered with *D. melanogaster* IRs (DmelIRs), *T. castaneum* IRs (TcasIRs) and *D. ponderosae* IRs (DponIRs) (Fig. 7). An amino acid sequence alignment of the putative *R. ferrugineus* iGluRs and IRs and *D. melanogaster* iGluRs and IRs identified variations in the characteristic glutamate binding residues at certain sub-regions of the S1 and S2 domains. In the S1 lobe, the arginine residue (R) was not well conserved, whereas in the S2 lobe, the threonine (T) residues at the start and the glutamic acid (E) or aspartic acid (D) residues at the end were moderately conserved (data not shown). In *R. ferrugineus*, we identified candidates for IR41a (contig 24327), IR21a (contigs 18505 and 26114), IR40a (contig 28605), and IR93a (contig 3182), as well as the coreceptors IR25a (contig 2728), IR76b (contig 5953) and IR8a (contig 32147 and RWP_IR_unigene 1). Two IR21a (contigs_18505 and 26114) isoforms were identified: contig_18505 clustered with DponIR21a and contig_26114 clustered with DmelIR21a. We assume that either IR21a is diverged within the Curculionidae family, or its candidate isoforms have not been reported in the *D. ponderosae* antennal transcriptome dataset [25]. Another candidate *R. ferrugineus* IR, RPW1_contig_28605, which shows identity with the DemlIR40a isoform G, has not been found in *D. ponderosae* and *I. typographus* [25]. In *R. ferrugineus*, IR41a, IR21a, IR68a and IR93a are extremely diverged from *T. castaneum* and *D. ponderosae*, with less than 30 % amino acid identity. Nevertheless, the coreceptors IR25a, IR8a, and IR76b are highly conserved and show more than 50–65 % identity with other coleopterans. Previous studies have reported on the dynamic patterns of expansion and divergence of IR families in both closely and distantly related species of insects occupying very different ecological niches [82].

A phylogenetic analysis revealed no apparent orthologous relationship between *R. ferrugineus* and *D. melanogaster* IRs or the three coleopteran (*T. castaneum*, *D. ponderosae* and *I. typographus*) IRs (Fig. 7). However, we found that *R. ferrugineus* IRs are more closely related to *T. castaneum*, *D. ponderosae* and *I. typographus* IRs and display a number of coleopteran-specific clades (Fig. 7). The IR subfamily consists of a set of conserved “antennal IRs” that likely define the first olfactory receptor family of insects. There are also species-specific “divergent IRs” that are expressed in the peripheral and internal gustatory neurons, and they are associated with taste and food assessment [82]. A reciprocal best-hit analysis and phylogenetic analysis of the *R. ferrugineus* and *Drosophila* IRs [17] identified several putative antennal IR orthologues in *R. ferrugineus*: IR68a, IR21a, IR40a, 93a and IR41a.

We searched for iGluRs-subfamily receptors in the *R. ferrugineus* antennal transcriptome dataset and found the iGluR, kainite and NMDA receptor subfamily genes, with most of them sharing more than 80 % amino acid identity with *T. castaneum*. In the *R. ferrugineus* antennal transcriptome, exhaustive BLASTx searches identified 12 iGluRs, 11 kainate receptor proteins and 5 NMDA receptor proteins. Interestingly, we did not find AMPA-subfamily transcripts in the *R. ferrugineus* antennal transcriptome. In *R. ferrugineus,* the iGluR-family is extremely divergent and exhibits an overall amino acid sequence identity of 5–79 % for iGluRs, 7–71 % for kainate receptors and 5–48 % for NMDA receptors. A phylogenetic analysis of the predicted iGluR, kainate and NMDA receptor protein sequences revealed that they are closely related to the members of the same clade (Coleoptera), and they share similarity with dipteran iGluR-subfamily receptors (Fig. 7). Among the iGluRs, 8 *R. ferrugineus* transcripts form a single clade putatively identified as IR75p (contigs_9708, 7823, 2193, 13244, 6943 and RPW_iGluR_unigene_2) and IR75d (contig_28514 and RPW_iGluR_unigene_1), which share 14-46 % amino acid identity. In *R. ferrugineus*, iGluR_contig_ 32388 is orthologous to DmeliGluR_IA and none of the coleopteran orthologous sequences are included this clade (Fig. 7). However, *R. ferrugineus* iGluR contigs 16595, 8388 and 30146, and orthologous sequences from *T. castaneum* form an identical clade (Fig. 7). In *R. ferrugineus*, 5 transcripts of the NMDA-subfamily were identified. Among these, contigs 32707 and 5838 clustered with *D. melanogaster* and *T. castaneum* NMDAs and share very low amino acid identity (>10 %). Hence, we can assume that they are highly diverged in insects. Three NMDA contigs (17407, 28307 and 24459) form a clade with *T. castaneum*, *D. ponderosae* and *I. typographus* and share more than 70 % amino acid identity. Hence, they are predicted to be specific to coleopterans (Fig. 7). Among the 11 kainate-subfamily members identified in *R. ferrugineus*, three closely related clades are proposed: two clades representing 9 contigs sharing high amino acid identity with the *Drosophila* kainate-subfamily and a third clade that is highly diverged from the *D. melanogaster* kainate family, with low amino acid identity (>10 %) (Fig. 7). In this study, we reported on several novel *R. ferrugineus* IRs and iGluRs-family receptors. Further studies combining coleopteran families are needed to reveal the evolution of this novel class of receptors in relation to the ecology of the species. These combination studies will ultimately help to develop novel targets for manipulating this newly discovered receptor family.

### Sensory neuron membrane proteins in *R. ferrugineus*

**Fig. 8:**
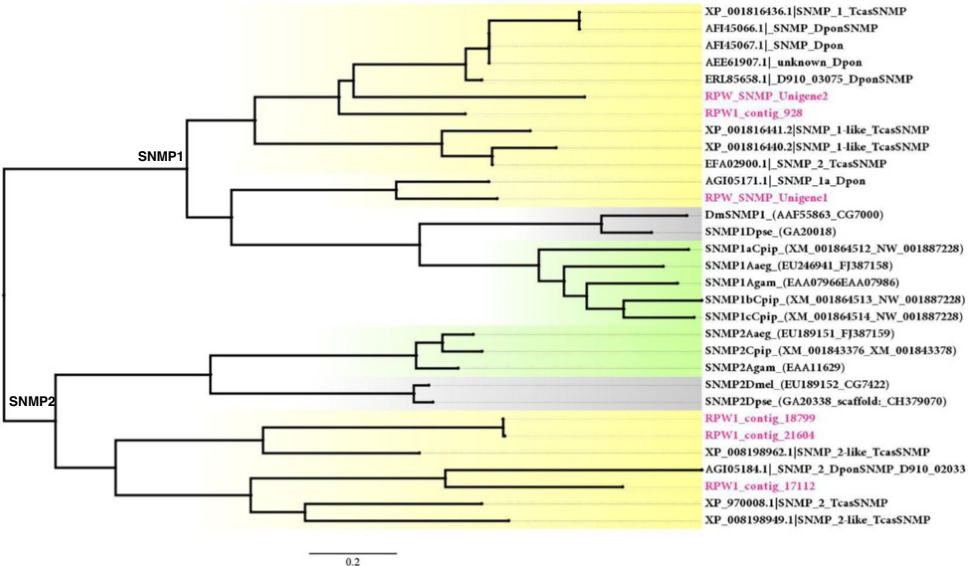
Unrooted tree of putative *R. ferrugineus* SNMPs with *D. melanogaster, D. pseudoobscura, Culex pipiens, A. gambiae, A. aegypti, T. castaneum* and *D. ponderosae* SNMPs. ML analysis was computed using MEGA (v.6.0) [42] (JTT model for ML heuristic searches methods was Nearest-Neighbor-Interchange). GenBank accession numbers are indicated. *R. ferrugineus* SNMPs are shown in red color. Lepidoptera, diptera and coleoptera clades are highlighted in yellow, grey and green color respectively. Scale represent 0.2 amino acid substitution per site

Sensory neuron membrane proteins (SNMPs) are a family of two trans-membrane domain proteins located in the dendrite membranes of odorant-sensitive ORNs, and they may function in the binding and transport of hydrophobic ligands [21, 86, 87]. Two SNMP subfamilies (SNMP1 and SNMP2) have been identified to date, and SNMP1 was found to be antenna-specific and was detected in pheromone-sensitive ORNs [88–90]. SNMP2 associates with pheromone-sensitive sensilla and is expressed in sensilla support cells, and it has been characterized from Lepidoptera [90]. In the present study, we reported six candidate SNMP-like genes in the *R. ferrugineus* antenna that all share an average 74 % amino acid identity (Additional file 9: Table S2). One SNMP transcript with a relatively high abundance of transcripts in the antennal transcriptome, RPW_unigene_1, appears to be expressed at high levels (cumulative RPKM: 1224) in the *R. ferrugineus* antenna and shares 69 % amino acid identity with *D. ponderosae* SNMP1 (Additional file 7: Figure S6E). Based on phylogenetic analysis and a BLASTx search, we identified three orthologous SNMP1- like proteins (RPW_SNMP_unigene 1, RPW1_contig_928 and RPW1_SNMP_unigene 2) that show identity with *T. castaneum* and *D. ponderosae* SNMP1, and three orthologous SNMP2-like proteins (RPW1_contig_21604, 17112 and 18799) that show similarity to *T. castaneum* and *D. ponderosae* SNMP2 (Fig. 8). In *R. ferrugineus*, SNMP1 transcripts (RPW1_contig_928 and RPW1_SNMP_unigene 2) appear to be highly conserved, with 88 % amino acid identity, compared with RPW1_SNMP_unigene 1 and RPW1_SNMP_unigene 2, which share 67 % identity (Additional file 9: Table S2). In addition, two SNMP2 transcripts (RPW1_contig_17112 and 18799) were found to share 77 % amino acid identity. A phylogenetic analysis of *R. ferrugineus* SNMPs with dipteran and lepidopteran insects shows a coleopteran-specific expansion of the SNMP genes (Fig. 8). Among the coleopterans, *R. ferrugineus* and *D. ponderosae* show an expansion of SNMP1 genes (Fig. 8). RPW_SNMP_Unigen_1 forms a cluster with *D. ponderosae,* and their amino acid alignment shows 70 % identity. RPW_SNMP_Unigene 2 and contig_928 clustered with *T. castaneum* and *D. ponderosae* SNMP1, showing 67–71 % amino acid identity. These results strongly suggest that *R. ferrugineus* SNMP1 originated from gene duplication events and remains distinct in this genus. In *R. ferrugineus*, SNMP2 contig 18,799 clustered with contig 21604, sharing 77 % amino acid identity, and it was found to be closely related to *T. castaneum* (XP_008198962) (Fig. 8). The proximity of these SNMP2 genes in Coleoptera suggests that they derived from gene duplication events.

The role of SNMPs in pheromone detection in Diptera and Lepidoptera antennae has been characterized [21, 22, 86, 88, 89]. The *Drosophila* homolog of SNMP1 is essential for the detection of the volatile pheromone 11-cis-vaccenyl acetate [86]. When *Hv*Cr13, the receptor for the main pheromone component (*Z*11-16:Ald) of *H. virescens,* was expressed in transgenic *Drosophila*, a SNMP protein was required for neuron responsiveness [21]*.* However*, Hv*Cr13 can respond to *Z*11-16:Ald in in-vitro assays lacking SNMPs [91]. In the moth, both SNMP1 and SNMP2 are expressed in the antennae and body parts [92]. We still lack information on the exact molecular function of the SNMP1 and 2 protein families, especially in Coleoptera. The large diversity of SNMP1 and 2 proteins within insect orders suggests that they contribute to the specificity of odour recognition [4]. In the present study, we identified three isoforms each of SNMP1 and SNMP2 in *R. ferrugineus*. Because SNMPs have key functions in chemoreception in Lepidoptera and Diptera, further studies on their functional characterization and a comparative analysis of *R. ferrugineus* SNMPs could significantly contribute to clarifying their important functions in Coleoptera.

### Gustatory receptors in *R. ferrugineus*

**Fig. 9:**
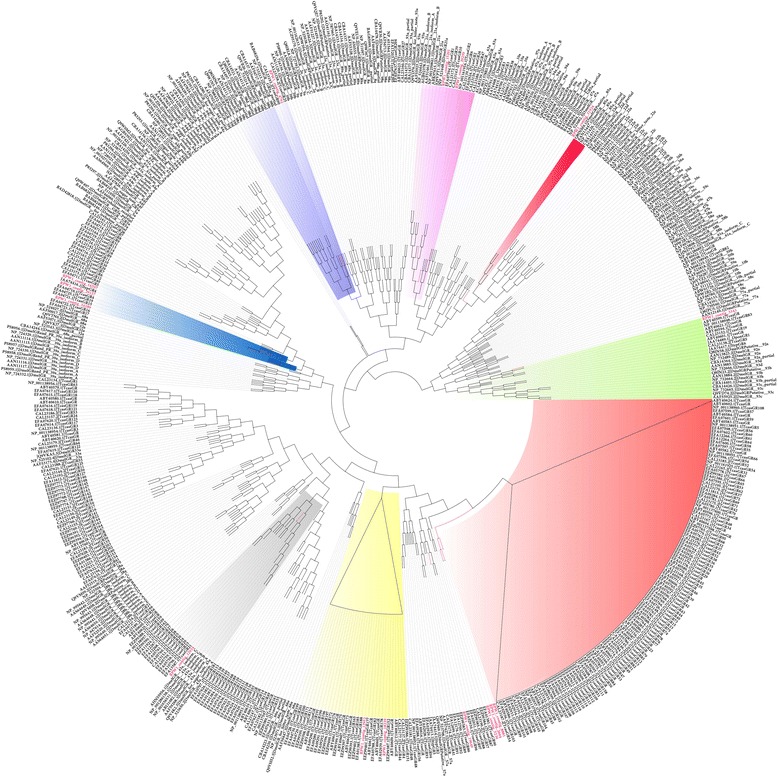
Unrooted tree of putative *R. ferrugineus* GRs constructed with those of *D. melanogaster*, *D. ponderosae, I. typographus* and *T. castaneum*. ML analysis was computed using MEGA (v.6.0) [42] (JTT model for ML heuristic searches methods was Nearest-Neighbor-Interchange). *R. ferrugineus* GR transcripts and node are marked with red color. *R. ferrugineus* GRs putatively identified as GR92a (contig 3343), 21a (contig 15201 and 29259), 98b (contig 4762), 68a (contig 16597, 29157 and 34189), 43a (contig 12934) and 85a (contig 31924) are highlighted in green, pink, purple, blue, gray and red color. *T. castaneum* specific GR clade is shown in peach color. Coleopteran specific clade close to Dmel66a is shown in yellow color. GenBank accession numbers are indicated. Scale represent 0.2 amino acid substitution per site. Refer Additional file 10: Table S3for the details of GenBank accession nos

Insect gustatory receptors (GRs) play an important role in the detection of taste chemicals and ultimately influence an insect’s decisions about food, mates and egg deposition sites [4, 93]. GRs belong to a highly conserved clade of insect proteins, and their functional characterization has been reported in *D. melanogaster* and *B. mori* [93, 94]. In addition, 62 uncharacterized GRs have been reported in *T. castaneum* [54, 70]. We identified 15 GRs in the *R. ferrugineus* antennal transcriptome with very low expression levels (RPKM > 10, with the exception of contigs 4762 and 5230) (Additional file 7: Figure S6F). Based on phylogenetic analysis, we discovered conserved insect-specific GRs orthologous to DmelGr21a (contigs 15201 and 29259), 92a (contig_3343), 43a (contig 12934), 68a (contigs 16597, 29157 and 34189), 85a (contig 31924) and 98b (contig 4762). A phylogenetic analysis of *R. ferrugineus*, *D. melanogaster* and *T. castaneum* GRs shows a coleopteran-specific expansion of GRs (Fig. 9). The phylogenetic tree also identified many closely related GRs in *T. castaneum*, which form a single clade. Similarly, three unique *R. ferrugineus* GRs (contigs 8881, 2604 and 5230), which form a single clade near the *T. castaneum* clade, were identified in the antennal transcriptome. The proximity of these *R. ferrugineus* GRs suggests that they are derived from gene duplication events. These trends indicate a dynamic pattern of the expansion and divergence of GRs in closely related species, and they also suggest that GRs contribute to the specificity of taste detection. One specific clade representing *R. ferrugineus* GRs (contigs 12952 and 18239) and several *T. castaneum* GRs is predicted to be coleopteran-specific and diverged from *D. melanogaster* GR66a and 57a. In the *R. ferrugineus* antenna, contig 31924 was found to be closely linked to *D. melanogaster* DmelGR85a, and its sequence has diverged from the dipteran clade. No representative GRs have been reported in *T. castaneum* and *D. ponderosae* (Fig. 9).

Insect GRs denote a highly divergent chemoreceptor family across all insects [95]. Limitations on investigating their functional characterization have led to a critical knowledge gap in this field. The reported functional characterizations of insect GRs include GR5a, GR61a, GR64a, e and f, and GR43a (sugar taste GRs) and GR21a/63a (CO_2_-sensing) from *D. melanogaster* [93], and GR8 and GR9 from *B. mori* (sugar taste) [94]. In the present study, we found orthologous sequences to isoforms 21a, 92a, 43a, 68a, 85a and 98b in the *R. ferrugineus* antenna as well as several species specific GRs. The large diversity of GRs in coleopterans suggests that they contribute to the specificity of taste detection, and they might have diverged from Diptera. Nevertheless, little is known about the specific functions of GRs in coleopterans*.* Further studies on the functional characterization and a comparative analysis of coleopteran GRs could significantly contribute to our knowledge about their important functions.

## Conclusions

The Red Palm Weevil, *Rhynchophorus ferrugineus*, is listed as a major invasive pest, and it is a serious threat to palm trees all over the world. We sequenced the *R. ferrugineus* antennal transcriptome, which, at 35,667 transcripts, represents the largest data set published to date for a coleopteran antennal transcriptome. We found great richness and diversity of *R. ferrugineus* OBPs and CSPs. Beetle-specific lineage expansions of ORs are direct indications of divergence, and they reveal the sophistication of the chemical communication systems in *R. ferrugineus*. We classified *R. ferrugineus* ORs into seven subfamilies of coleopteran ORs and predicted two new subfamilies. We also identified putative aggregation pheromone receptors from *R. ferrugineus* that grouped into a unique beetle clade. Several highly diverged IR orthologues were identified, including unique GR orthologues for CO_2_-sensing and sweet tastants, as well as a highly expressed SNMP isoform. This study provides strong background information on novel olfactory multi-gene families, and it may provide potential targets for disrupting the chemical communication system in *R. ferrugineus* as a means of pest control. The data will be useful for functional characterization studies that will ultimately facilitate a better understanding of the behaviour of *R. ferrugineus*, and the information will enable a more informed approach to the management of this quarantine pest that inflicts significant damage each year to palm trees throughout the world.

## Additional files

Additional file 1: Figure S1. Characteristics of homology searches of *R. ferrugineus* protein-coding genes against the non-redundant protein sequences (*nr*) at NCBI using BLASTp. (DOCX 139 kb) Additional file 2: Figure S2. Mapping of *R. ferrugineus* protein-coding genes to GO terms associated to BLASTp hits. (DOCX 106 kb) Additional file 3: Figure S3. Functional assignment of terms to query sequences from the pool of GO terms gathered in the mapping step. (DOCX 181 kb) Additional file 4: Figure S4. Distribution of enriched functions in A) Biological Process (BP), B) Cellular Component (CC) and C) Molecular Functions (MF). (DOCX 263 kb) Additional file 5: Table S1. The highly conserved genes among *R. ferrugineus, D. ponderosae* and *I. typographus.* (XLSX 130 kb) Additional file 6: Figure S5. Alignment of the *R. ferrugenieus* OBPs contigs. (DOCX 59 kb) Additional file 7: Figure S6 (A-F). The relative abundances of different chemosensory gene families in the *R. ferrugineus* antennal transcriptome dataset, presented as reads per kilobase per million reads (RPKM). (DOCX 52 kb) Additional file 8: Figure S7. A family of divergent odorant binding proteins (OBP) in *R. ferrugineus.* (DOCX 63 kb) Additional file 9: Table S2. Percent identity of insect SNMPs. (XLSX 13 kb) Additional file 10: Table S3. GenBank accession numbers of the GR and OR proteins used for the tree construction (Figures 9 and 5). (XLSX 25 kb)

## Abbreviations

CSP: Chemosensory protein
GO: Gene ontology
GR: Gustatory receptor
IR: Ionotropic receptor
MSA: Multiple sequence alignment
OBP: Odorant binding protein
OR: Odorant receptor
Orco: OR-coreceptor
ORNs: Olfactory receptor neurons
OSN: Olfactory sensory neuron
PR: Pheromone receptor
RPKM: Reads per kilobase of transcript per million mapped reads
RPW: Red palm weevil
SNMP: Sensory neuron membrane protein
