# Supplementary material for: Identification of the genes involved in odorant reception and detection in the palm weevil Rhynchophorus ferrugineus, an important quarantine pest, by antennal transcriptome analysis
Source: BMC Genomics. 2016 Jan 22;17:69. doi: 10.1186/s12864-016-2362-6 (PMC4722740; doi:10.1186/s12864-016-2362-6)

**Additional file 1: Figure S1. Characteristics of homology searches of *R. ferrugineus* protein-coding genes against the non-redundant protein sequences (*nr*) at NCBI using BLASTp.** **(A)** e-value distribution of the top BLAST hit for each unique sequence with a cut-off e-value of 1.0E-6. **(B)** Similarity distribution of the top BLAST hit for each unique sequence. The sequence similarity of *R. ferrugineus* with database by Blast search ranges from 36% to approx. 100% and **(C)** Top-species distribution of the top BLAST hit for each unique sequence. The sequences of *R. ferrugineus* sequences showed the most significant similarity to the sequences of *D. ponderosae* followed by sequences of *T. castaneum*.


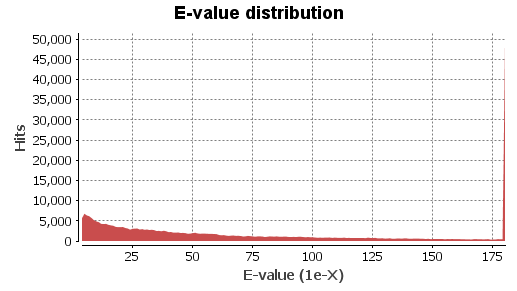


**A**


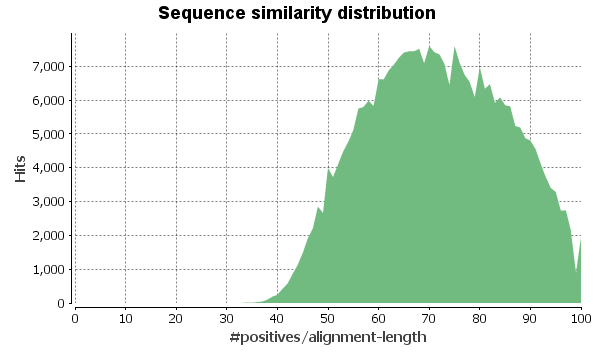


**B**

**C**


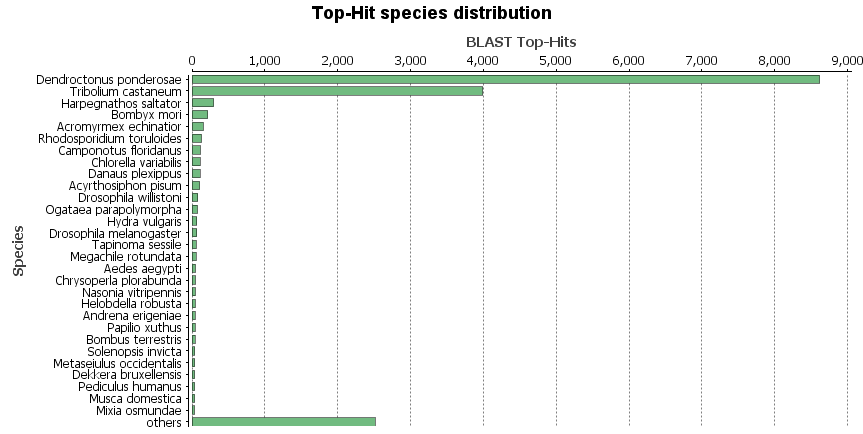

Supplement: Additional file 1: Figure S1. — Characteristics of homology searches of R. ferrugineus protein-coding genes against the non-redundant protein sequences (nr) at NCBI using BLASTp. (DOCX 139 kb) [file 12864_2016_2362_MOESM1_ESM.docx]
