# Supplementary material for: Identification of the genes involved in odorant reception and detection in the palm weevil Rhynchophorus ferrugineus, an important quarantine pest, by antennal transcriptome analysis
Source: BMC Genomics. 2016 Jan 22;17:69. doi: 10.1186/s12864-016-2362-6 (PMC4722740; doi:10.1186/s12864-016-2362-6)

**Additional file 2: Figure S2. Mapping of *R. ferrugineus* protein-coding genes to GO terms associated to BLASTp hits.** **(A)** Evidence code distribution for BLAST hits. The evidence code distribution for BLAST hits chart shows an overrepresentation of Inferred Electronic Annotation (IEA), followed by Inferred by Mutant Phenotype (IMP); **(B)** Evidence code distribution for individual sequences. The highest evidence code for the individual sequences was through Inferred Electronic Annotation (IEA), second by Inferred by Mutant Phenotype (IMP) and third by Inferred by Direct Assay (IDA); **(C)** Mapping database sources. The majority of *R. ferrugineus* genes are obtained from the UniProt Knowledge Base (KB) followed by FlyBase (FB).


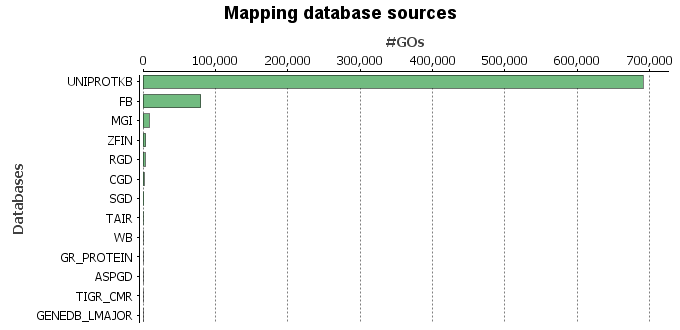


**B**

**C**

**A**


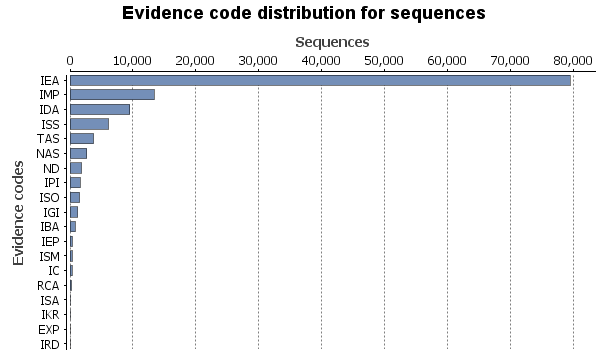


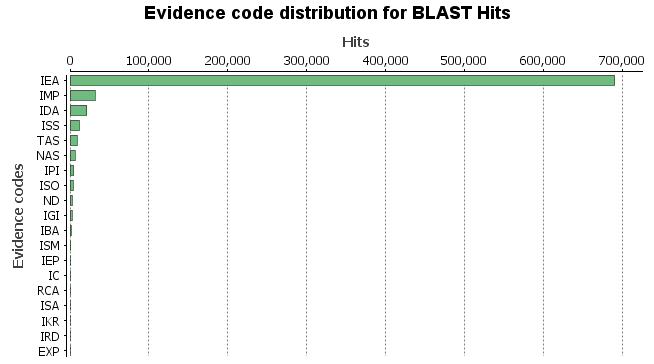

Supplement: Additional file 2: Figure S2. — Mapping of R. ferrugineus protein-coding genes to GO terms associated to BLASTp hits. (DOCX 106 kb) [file 12864_2016_2362_MOESM2_ESM.docx]
