# Supplementary material for: Identification of the genes involved in odorant reception and detection in the palm weevil Rhynchophorus ferrugineus, an important quarantine pest, by antennal transcriptome analysis
Source: BMC Genomics. 2016 Jan 22;17:69. doi: 10.1186/s12864-016-2362-6 (PMC4722740; doi:10.1186/s12864-016-2362-6)

**Additional file 3: Figure S3. Functional assignment of terms to query sequences from the pool of GO terms gathered in the mapping step.** **(A)** Data distribution with 30,582 blast hits, 53,210 without blast hit, 4,217 with mapping results and 20,615 annotated sequences **(B)** Annotation distribution; Most sequences have between 1 and 6 GO terms annotated; **(C)** GO-level distribution. *R. ferrugineus* sequence GO terms representation for biological process (BP), molecular function (MF) and cellular component (CC) ontologies. The mean GO-level is 4.385 and 97437 annotations could be assigned; **(D)** Number of GO-terms for *R. ferrugineus* sequences with length (x). The length of most GO term annotated *R. ferrugineus* sequences with average 857 bp; **(E)** Annotation score distribution; and **(F)** Percentage of *R. ferrugineus* sequences with length (x) annotated.

**B**

**A**


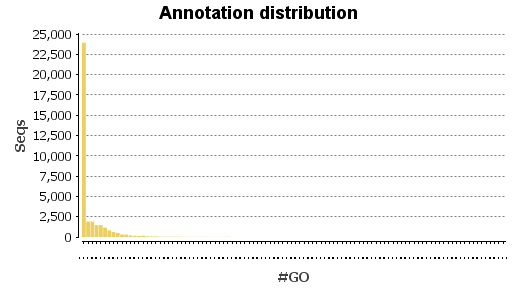


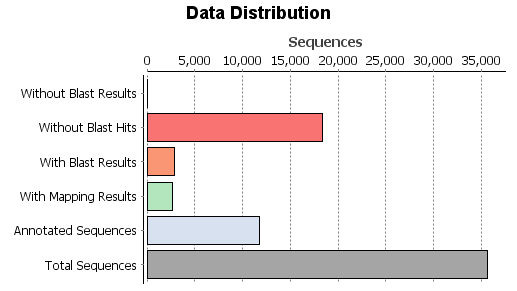


**C**


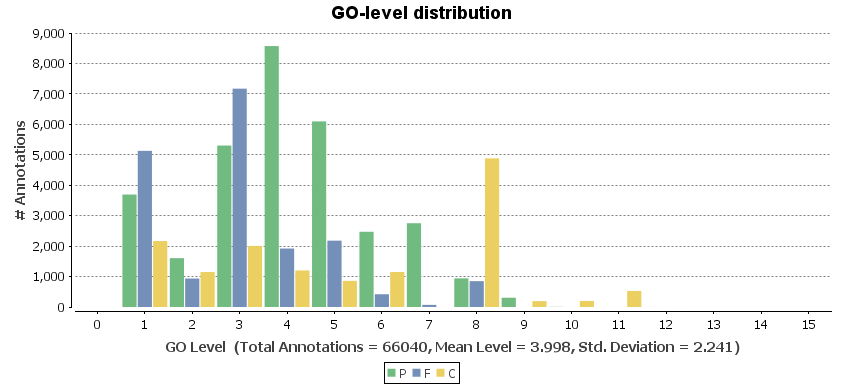


**D**.


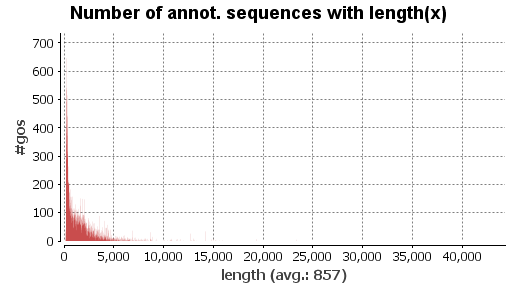


P: Biological process, F: Molecular function; C: cellular component

**F**

**E**


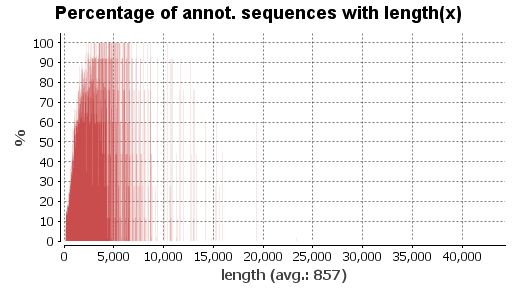


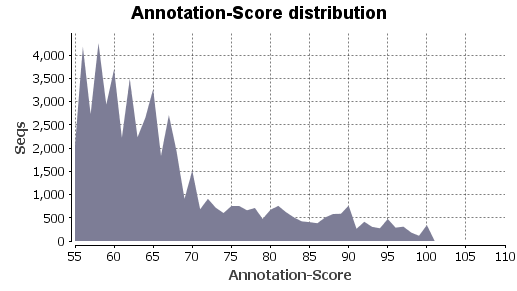

Supplement: Additional file 3: Figure S3. — Functional assignment of terms to query sequences from the pool of GO terms gathered in the mapping step. (DOCX 181 kb) [file 12864_2016_2362_MOESM3_ESM.docx]
