# Supplementary material for: Identification of the genes involved in odorant reception and detection in the palm weevil Rhynchophorus ferrugineus, an important quarantine pest, by antennal transcriptome analysis
Source: BMC Genomics. 2016 Jan 22;17:69. doi: 10.1186/s12864-016-2362-6 (PMC4722740; doi:10.1186/s12864-016-2362-6)
Supplement: Additional file 4: Figure S4. — Distribution of enriched functions in A) Biological Process (BP), B) Cellular Component (CC) and C) Molecular Functions (MF). (DOCX 263 kb) [file 12864_2016_2362_MOESM4_ESM.docx]

**Additional file 4: Figure S4. Distribution of enriched functions in A) Biological Process (BP), B) Cellular Component (CC) and C) Molecular Functions (MF).** For each panel, the chart displays the distribution of annotated sequences (50 nos) by BP, MF, or CC in *R. ferrugineus*.


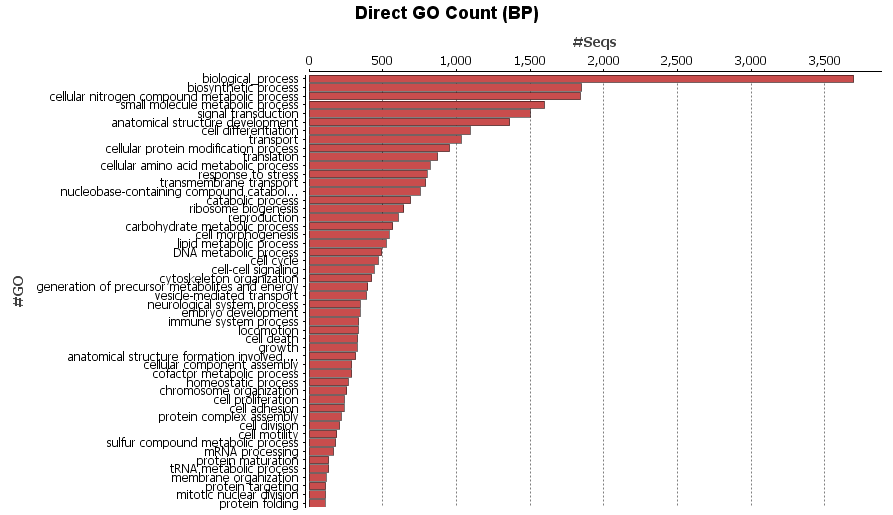


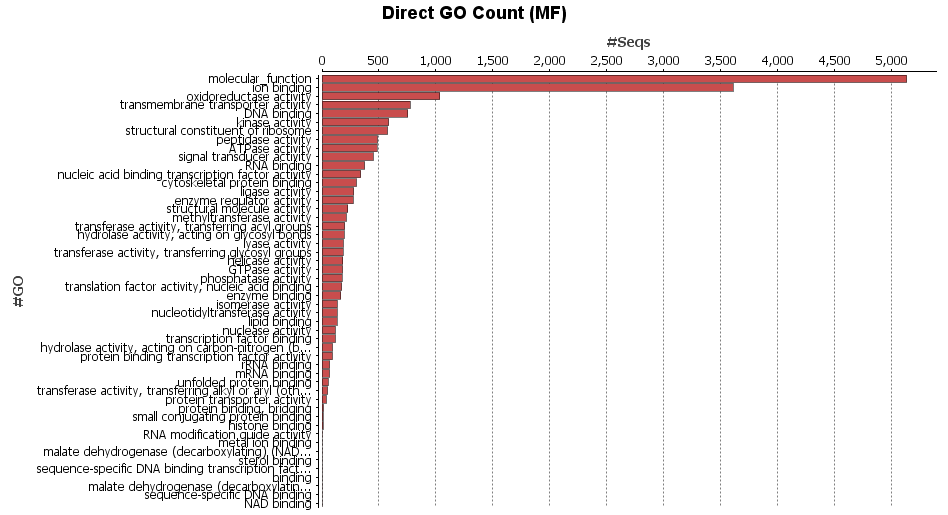


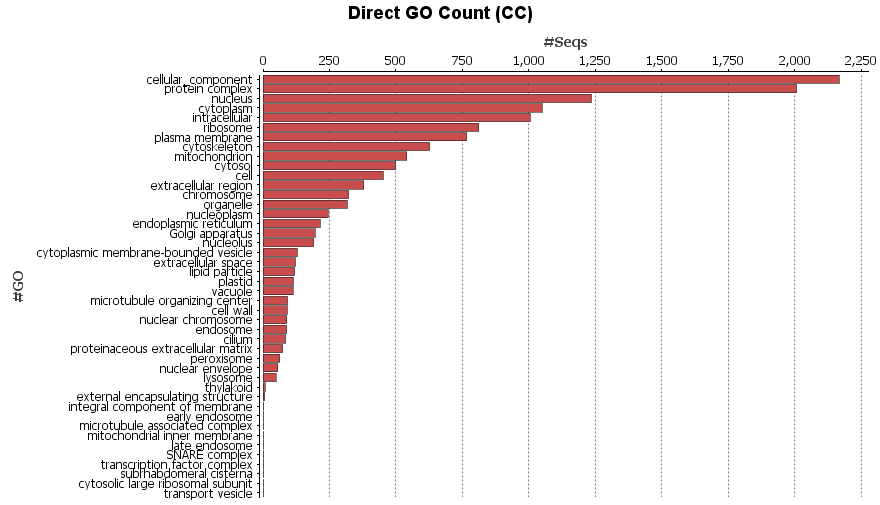


**A**

**B**

**C**
