# Supplementary material for: Identification of the genes involved in odorant reception and detection in the palm weevil Rhynchophorus ferrugineus, an important quarantine pest, by antennal transcriptome analysis
Source: BMC Genomics. 2016 Jan 22;17:69. doi: 10.1186/s12864-016-2362-6 (PMC4722740; doi:10.1186/s12864-016-2362-6)
Supplement: Additional file 6: Figure S5. — Alignment of the R. ferrugenieus OBPs contigs. (DOCX 59 kb) [file 12864_2016_2362_MOESM6_ESM.docx]

**Additional file 6: Figure S5. Alignment of the *R. ferrugenieus* OBPs contigs.** Highly conserved cysteine are shown with dark arrowhead above. Conserved residues are shown in hollow arrowheads.

**C1**

10 20 30 40 50 60 70 80 90 100

....|....| ....|....| ....|....| ....|....| ....|....| ....|....| ....|....| ....|....| ....|....| ....|....|

**RPW1_contig_23**  **----------** **----------** **----------** **----------** **----------** **----------** **-DEMKELAAQ** **------LHNA** **C---------** **-----V--AE**

**RPW1_contig_23127** **----------** **----NVLAI-** **----------** **----------** **----------** **------SDEE** **KAAIKESVLK** **------YVQE** **C---------** **-----G--QE**

**RPW1_contig_77**  **----------** **---MKTFIVL** **SAFVL-----** **----------** **----------** **------AVVL** **GATTKSSWNS** **------VHQA** **C---------** **-----QAKPG**

**RPW1_contig_107**  **----------** **--KFD-----** **----------** **----------** **----------** **------DSII** **SEDIRKLLKG** **------LHDV** **C---------** **-----V--SK**

**RPW1_contig_382**  **----------** **---LTMKVLC** **LAIFVFA---** **----------** **----------** **------TVVM** **ADHVQVRYDN** **------VHKN** **C---------** **-----QKDPA**

**RPW1_contig_446**  **----------** **----------** **----------** **----------** **----------** **------CQEF** **TEEQKKKILE** **------NRKQ** **C---------** **-----I--EE**

**RPW1_contig_1768**  **----------** **----------** **----------** **----------** **----------** **---------M** **TPEQRTRFFN** **------FQNE** **C---------** **-----M--QE**

**RPW1_contig_3937**  **----------** **--MKYLIVLV** **LSLSAIT---** **----------** **----------** **------AKPL** **TDEQKQNFRN** **------FYDG** **C---------** **-----V--SE**

**RPW1_contig_3997**  **----------** **--MKQVLALV** **LCV-------** **----------** **----------** **------SVVM** **GQSVLEKWEK** **------LHDV** **C---------** **-----QSDPA**

**RPW1_contig_4661**  **----------** **----------** **----------** **----------** **----------** **----------** **----------** **-------KEV** **INTCQDEIKI** **VILSEAL-EA**

**RPW1_contig_11442** **----------** **----------** **----------** **----------** **----------** **----------** **----------** **---------E** **T---------** **--------TR**

**RPW1_contig_12010** **----------** **----------** **----------** **----------** **----------** **------VASS** **KKHQKNNEVT** **---PKKAFKE** **C---------** **-----QKNAA**

**RPW1_contig_14025** **----------** **-------ALV** **LCTWLLDYVQ** **PAP-------** **----------** **------PPAF** **QLPSEEERNR** **------IALK** **C---------** **-----I--DE**

**RPW1_contig_23691** **----------** **--MFKINLFT** **LLFISIT---** **----------** **----------** **------TADL** **VANRKDATLK** **------PLSA** **C---------** **-----CDIPE**

**RPW1_contig_28119** **----------** **----------** **----------** **----------** **----------** **----------** **--ESQEKLKQ** **------AHQK** **C---------** **-----QSDPA**

**RPW1_contig_29381** **----------** **----------** **----------** **----------** **----------** **----------** **------RYFQ** **------AHEQ** **C---------** **-----QSNPE**

**RPW1_contig_33721** **----------** **----------** **----------** **----------** **----------** **----------** **----------** **----------** **----------** **----------**

**RPW1_contig_10788** **----------** **----------** **----------** **YT--------** **----------** **------MADL** **SDEQKQKVVG** **------YGRE** **C---------** **-----I--AE**

**RPW1_contig_12481** **----------** **----------** **----------** **----------** **----------** **----------** **----------** **----------** **----------** **----------**

**RPW1_contig_12511** **----------** **----------** **----------** **----------** **----------** **---------L** **TPEQQEAKLE** **------SQRH** **C---------** **-----L--KE**

**RPW1_contig_14511** **----------** **--MNTLLALC** **VILASACVVT** **TAYDFEDAEF** **NQILADDLED** **GLDTLDSTFV** **HHRVRRAEDT** **NPSPQNSDEK** **CKKKRRKPSL** **CCADDIFEQE**

**RPW1_contig_16551** **----------** **----------** **----------** **----------** **----------** **----------** **----------** **------FHNE** **C---------** **-----L--NE**

**RPW1_contig_1689**  **----------** **----------** **----------** **----------** **----------** **---------M** **TPEQRQRFLT** **------FQGE** **C---------** **-----M--ME**

**RPW1_contig_17793** **----------** **--MQATFTLL** **VIIGYNAIS-** **----------** **----------** **------ALTL** **LPEEKQFGDE** **------VVKQ** **C---------** **-----I--TE**

**RPW1_contig_19755** **----------** **--MHNFTIIV** **ILACTFVSAL** **A---------** **----------** **------VGDL** **TPEQRQRFFN** **------FQNE** **C---------** **-----M--QE**

**RPW1_contig_2374**  **----------** **----------** **--------YV** **----------** **----------** **------SAEL** **SEEQKQKVVS** **------YGKE** **C---------** **-----I--SD**

**RPW1_contig_257**  **----------** **----LIFSVC** **LLVSLLQ---** **----------** **----------** **------VKCQ** **TDKQKELLAQ** **------HYKH** **C---------** **-----V--EE**

**RPW1_contig_29**  **----------** **----MTVILV** **SIFLVFVANT** **IA--------** **----------** **------KVTL** **PPELQEYVDE** **------IHSV** **C---------** **-----L--SK**

**RPW1_contig_3199**  **----------** **----------** **----------** **----------** **----------** **---NGLSESL** **IDEMKEKVQT** **------FGLE** **C---------** **-----A--QQ**

**RPW1_contig_3213**  **----------** **----------** **----------** **----------** **----------** **------VKAM** **SDEMKELAQM** **------LHNT** **C---------** **-----V--GE**

**RPW1_contig_7073**  **----------** **---MKQFLLI** **ITVIGISSTV** **----------** **----------** **------FAEL** **SEAQKQKVDS** **------YENE** **C---------** **-----M--VK**

**RPW1_contig_8586**  **----------** **----------** **----------** **----------** **----------** **----------** **----------** **----------** **C---------** **-----QGIGD**

**RPW1_contig_9136**  **----------** **----------** **----------** **----------** **----------** **-------AET** **SDERKQKIAA** **------FTNH** **C---------** **-----I--VE**

**RPW1_contig_981**  **----------** **---MKSFLLV** **TVVISLVLYV** **----------** **----------** **------SAEL** **SEEQKQKVVS** **------YGKD** **C---------** **-----I--AD**

**RPW1_contig_9915**  **----------** **--KMYIFTLI** **VIFGIGSIAA** **NQQK------** **----------** **------LVHL** **TADKLYQILP** **------HIMK** **C---------** **-----A--DE**

**RPW_OBP_Unigene_1** **----------** **------FLVL** **VGSVIKL---** **----------** **----------** **------TDAQ** **RDPQRQKIID** **------FHAE** **C---------** **-----L--DA**

**RPW_OBP_Unigene_2** **----------** **----------** **----------** **----------** **----------** **-------ADL** **SDEQKQKVVA** **------FGKE** **C---------** **-----I--AE**

**RPW_OBP_Unigene_3** **----------** **---MKSFLVI** **SSVLCMSIYV** **----------** **----------** **------LADL** **SDEQKQKIIS** **------YGKE** **C---------** **-----I--AE**

**C3**

**C2**

110 120 130 140 150 160 170 180 190 200

....|....| ....|....| ....|....| ....|....| ....|....| ....|....| ....|....| ....|....| ....|....| ....|....|

**RPW1_contig_23**  **TGATEDA---** **-ITN---AR-** **-----AGTF-** **-AD--DD---** **NFKCYFK-CL** **FDQMA-----** **----------** **------IMDD** **EG-IIDVEAM** **IAVLPDEYQD**

**RPW1_contig_23127** **YDISEEK---** **-LKE---AH-** **-----EKKS-** **-SDGIEP---** **---CAIG-CI** **FKKAS-----** **----------** **------FING** **QG-LFDAEKA** **KQIGTKYIKG**

**RPW1_contig_77**  **VFVDDAI---** **-FEK---LK-** **-----RGEK-** **-VQ--LP--A** **NFGVHAH-CM** **LEGFG-----** **----------** **------IQNS** **QG-AIQQSGV** **KKAVQESVSD**

**RPW1_contig_107**  **TAVDEVL---** **-IEK---LK-** **-----DAEF-** **-TE--DQ---** **KLKCYVQ-CL** **LVQTG-----** **----------** **------SMDL** **AG-HIDIEAA** **VELIPEQIRN**

**RPW1_contig_382**  **LYVDDAI---** **-FAK---LK-** **-----KGEK-** **-VDNLPA---** **NFGAHAF-CM** **LKNLD-----** **----------** **------LQDG** **QG-KIQGAGV** **QKAVEKSITD**

**RPW1_contig_446**  **TKVNPEL---** **-IEK---AD-** **-----QGNF-** **-VD--DN---** **SLKCFTK-CF** **YQKAG-----** **----------** **------FVND** **EG-EVQLDVV** **KAKLPPQADK**

**RPW1_contig_1768**  **TGATDEM---** **-VLK---AF-** **-----AGEL-** **-TD--SP---** **VFKDHLV-CV** **GMKDGTESVR** **WRTHRLTCVQ** **RSSGLCWYEX** **XX-XXXXXXX** **XKGIMLFVDD**

**RPW1_contig_3937**  **TNVDKAV---** **-VEK---AH-** **-----HGEF-** **-EN--DD---** **KLPFFFL-CM** **SKKIG-----** **----------** **------FRDA** **NN-VFQPSSI** **KEK-------**

**RPW1_contig_3997**  **TFVDESI---** **-FEK---VK-** **-----NNET-** **-VEL-PP---** **NFGAHVF-CM** **TVNLN-----** **----------** **------IQDP** **NG-KFNKEVT** **AKLIGEVVKD**

**RPW1_contig_4661**  **LNINEEK---** **--SR---AK-** **-----RSVFS** **PDE--KR---** **IAGCLLQ-CV** **YRKLE-----** **----------** **----------** **----------** **--AXXXYVTQ**

**RPW1_contig_11442** **APISDDA---** **-LEK---TL-** **----------** **-SD--KR---** **YLQRQLK-CA** **LGEAP-----** **----------** **-------CDP** **VG-----RRI** **KSIAPLVLRG**

**RPW1_contig_12010** **TRIDKQA---** **-VKK---YK-** **-----KKEV-** **-----DTMPQ** **NYGEHLL-CV** **YKAIG-----** **----------** **------LIGE** **NG-TVNQDAL** **KKKISKKAQA**

**RPW1_contig_14025** **VKIEKSI---** **-IEE---VL-** **-----KTQVL** **PHD--DK---** **KYKKFLE-CS** **YRKQG-----** **----------** **------FLSL** **DGSRMLYDNL** **FLFLTEFYNI**

**RPW1_contig_23691** **LGDPKPL---** **-------SE-** **-----CSNP-** **-----KP---** **PGPCNDIQCA** **FEKSG-----** **----------** **------FLKD** **KN-TLNKEAY** **KXXXPKIYDD**

**RPW1_contig_28119** **TAVEEAS---** **-LKG---LA-** **-----RGGP-** **-----KP--A** **NYGAHSL-CI** **SKALG-----** **----------** **------WQNE** **DG-SVNADTI** **RARAENIFGS**

**RPW1_contig_29381** **TCMDEKE---** **-FRC---IK-** **--RRENGVL-** **-----TA---** **NFTRHAA-CM** **FKRLG-----** **----------** **------FLSD** **EG-QLQKDTM** **GQVIAWNYKD**

**RPW1_contig_33721** **-GVNDDD---** **-YEL---IK-** **-----KKKV-** **-PS--SP---** **EGICMVD-CL** **FTKLH-----** **----------** **------IIEH** **-G-RFNKRGF** **VVIFSAAVRG**

**RPW1_contig_10788** **TGVEKEL---** **-VSK---AR-** **-----QGSF-** **-SD--DP---** **KLKAFAF-CL** **SKKIG-----** **----------** **------LQNA** **SG-DVQADVI** **KQKLASIVNN**

**RPW1_contig_12481** **----------** **----------** **----------** **------P---** **EFKKHLL-CV** **KKAAG-----** **----------** **------IVDD** **RG-NYNKDAL** **RDTISTYVDD**

**RPW1_contig_12511** **TGATEEM---** **-VRN---AF-** **-----KGEI-** **-SD--AP---** **EFKKHLF-CV** **KKSAG-----** **----------** **------IVDD** **SG-NYNKDVL** **RETVSKYVDD**

**RPW1_contig_14511** **HEKDRDIFRS** **CFREVLGVEK** **SSHHRRGDPF** **SCKETEKRRN** **DMTCVAA-CC** **GQKKG-----** **----------** **------LLDD** **QG-QPKPEAI** **TKAIKEAFAK**

**RPW1_contig_16551** **SNLTTDE---** **-LQE---QK-** **------TQL-** **-SE--RK---** **--LCYIK-CM** **ALKVG-----** **----------** **------FLLR** **DG-TVLINKA** **REMLPRKIFT**

**RPW1_contig_1689**  **SGTTEEM---** **-LLK---AF-** **-----MGEF-** **-TE--SS---** **VFKDHLV-CL** **GMKTG-----** **----------** **------VIDD** **EG-NYHKDIL** **KEEILSFIGD**

**RPW1_contig_17793** **TSISKSI---** **-LEM---HE-** **----------** **-IN--DENRE** **KAGSFAL-CI** **SKKVG-----** **----------** **------YQDD** **KG-NLQTDDI** **RKVLTSIIGN**

**RPW1_contig_19755** **TGATDDM---** **-ILK---AF-** **-----RGEL-** **-TD--SP---** **LFKDHLV-CL** **GLKSG-----** **----------** **------VVDK** **EG-NYHKEVL** **KKEIMMFTDD**

**RPW1_contig_2374**  **TGVDKEI---** **-VHK---AH-** **-----EGSF-** **-SD--DP---** **KLKAFAF-CM** **SKKIG-----** **----------** **------LQSD** **SG-EVQTEVL** **KQKLSSVVDD**

**RPW1_contig_257**  **THVDQNV---** **-LQQ---AR-** **-----AGNF-** **-TD--DP---** **KLKDHIL-CI** **TKKIG-----** **----------** **------FQDD** **AG-HLQKEVI** **QKKLKEAVKG**

**RPW1_contig_29**  **SGLTEDH--H** **SAYD---IK-** **----------** **-HK--DE---** **KMMCYMK-CL** **MLESK-----** **----------** **------WMTT** **DG-TIDYEFI** **EAQSYPEVKD**

**RPW1_contig_3199**  **EKATEED---** **-IQA---LL-** **-----KKQA-** **-PV--TH---** **GGKCTIF-CT** **MKKFD-----** **----------** **------LMKE** **DG-SFGPGDL** **DWIERAKADD**

**RPW1_contig_3213**  **TGVSEDL---** **-IEK---VN-** **----SAKTF-** **-AD--DE---** **NLKCYIK-CL** **MAQMA-----** **----------** **------CIDD** **DG-IIDVEAT** **IAVLPEEIQQ**

**RPW1_contig_7073**  **TGVARDV---** **-VEK---AQ-** **-----QGTL-** **-ID--DP---** **KLKAFAF-CI** **SKKIG-----** **----------** **------LQNE** **KG-EVQIEAL** **KEQLPSLVEN**

**RPW1_contig_8586**  **SKIDEEV---** **-FQK---LD-** **-----QNEP-** **-VDL-PP---** **NFGKHML-CM** **MQGIG-----** **----------** **------AVTS** **DG-HISQDGV** **KTHIRHVVSD**

**RPW1_contig_9136**  **TGIDGNI---** **-VQN---AL-** **-----QGNI-** **-VE--DP---** **KLKTFAF-CM** **TKKAG-----** **----------** **------LQNA** **NG-DVQIEEL** **KKQLPNLVDN**

**RPW1_contig_981**  **TGVDKEI---** **-VQK---AH-** **-----QGSF-** **-SD--DP---** **KLKAFAF-CM** **SKKIG-----** **----------** **------LQSE** **SG-DVQTDVL** **KQKLSSVVDD**

**RPW1_contig_9915**  **TNVNFEE---** **-LRY---LG-** **-----TDKGD** **-RG--DR---** **NVGGFIS-CS** **FKRTG-----** **----------** **------YADE** **MG-NVKVGKF** **LQLFPIEYQE**

**RPW_OBP_Unigene_1** **HGVDEEA---** **-MIE---AL-** **-----DGHP-** **-PD--DD---** **AFYMHLF-CA** **AKKAK-----** **----------** **------VMDE** **NG-TVNVDNF** **HIDMAHVIDE**

**RPW_OBP_Unigene_2** **TGVDKAL---** **-VEK---AR-** **-----QGSF-** **-SD--DP---** **KLKAFAF-CL** **SKKIG-----** **----------** **------LQNA** **SG-DVQVDVL** **KQKLSSVVSS**

**RPW_OBP_Unigene_3** **TGVDKEL---** **-VDK---AR-** **-----QGSF-** **-SD--DP---** **KLKAFAF-CL** **SKKIG-----** **----------** **------LQNA** **DG-DVQAEVL** **KEKLSSVVDN**

**C6**

**C5**

**C4**

210 220 230 240 250 260 270 280 290 300

....|....| ....|....| ....|....| ....|....| ....|....| ....|....| ....|....| ....|....| ....|....| ....|....|

**RPW1_contig_23**  **--------TL** **PPVIRKCDTK** **K---------** **--GAN--PCE** **NA--------** **--WL-THK-C** **YY--------** **---------Q** **ENPAHYFLI-** **----------**

**RPW1_contig_23127** **EEDR---KKY** **DEIADEC---** **----------** **----------** **----------** **----------** **----------** **----------** **----------** **----------**

**RPW1_contig_77**  **ASKV------** **NQIVSACSVS** **----------** **--KGT--KEA** **TA--------** **--LE-IFK-C** **F---------** **----------** **----------** **----------**

**RPW1_contig_107**  **--------AV** **IKDVNKCAKD** **SEQ-------** **--VAE--HCD** **RA--------** **--FA-TLK-C** **LY--------** **---------S** **VNPDIYYVF-** **----------**

**RPW1_contig_382**  **QAKA------** **KQVTAECSGV** **N---------** **--KGT--KED** **TA--------** **--LA-LFD-C** **FG--------** **---------K** **H---------** **----------**

**RPW1_contig_446**  **EQAL------** **-AIVEKCKIK** **----------** **--GKD--ACD** **TV--------** **--YL-IHK-C** **YF--------** **---------E** **HTHP------** **----------**

**RPW1_contig_1768**  **EGKV------** **DAMLDKCYTH** **----------** **--YDT--QQD** **TA--------** **--FN-MMK-C** **MF--------** **---------K** **EH--------** **----------**

**RPW1_contig_3937**  **----------** **----------** **----------** **----------** **----------** **----------** **----------** **----------** **----------** **----------**

**RPW1_contig_3997**  **QAKV------** **NKIVNECAVN** **----------** **--KPN--KDD** **AA--------** **--VA-FLQ-C** **LD--------** **---------K** **NNV-------** **----------**

**RPW1_contig_4661**  **KEYV---LAT** **RQAVNTCLTN** **VQKTYSVTPH** **SIQES-KVCE** **VA--------** **--YD-VFE-C** **VS--------** **----------** **----------** **----------**

**RPW1_contig_11442** **SCPQ------** **------CTDK** **----------** **--EQK--QIK** **KV--------** **--LA-YVQ-V** **NY--------** **--------PK** **EWNKMLQQYA** **S---------**

**RPW1_contig_12010** **GQNV------** **DTLLQECGAA** **----------** **--KAD--PKQ** **TA--------** **--ID-MDS-C** **LI--------** **---------K** **NNL-------** **----------**

**RPW1_contig_14025** **DD--------** **LDALEHCKYI** **K---------** **--SKD--PGD** **LC--------** **--FQ-NLS-C** **IL--------** **---------D** **ALRTVE----** **----------**

**RPW1_contig_23691** **ED--------** **----------** **----------** **----------** **----------** **----------** **----------** **----------** **----------** **----------**

**RPW1_contig_28119** **SAKL------** **DEVISECAQN** **----------** **--QAN--AEE** **TA--------** **--IH-LTR-C** **YA--------** **---------K** **Y---------** **----------**

**RPW1_contig_29381** **PKKV------** **MAILNECYSN** **----------** **--KAT--KEE** **TS--------** **--LG-VFH-C** **FR--------** **---------K** **N---------** **----------**

**RPW1_contig_33721** **NIQK--LKKL** **NDLGNMCEKE** **I---------** **----------** **----------** **----------** **----------** **----------** **----------** **----------**

**RPW1_contig_10788** **ADSI------** **NNLISACVQK** **----------** **--KAT--PEE** **TA--------** **--FH-TFV-C** **YY--------** **---------E** **NTPNHVSIF-** **----------**

**RPW1_contig_12481** **------TDTI** **NSMIEKCFKM** **----------** **--MDS--PEE** **TA--------** **--YQ-MTK-C** **F---------** **----------** **----------** **----------**

**RPW1_contig_12511** **ADTV------** **NSMIEKCFKM** **----------** **--MES--PEE** **TA--------** **--YE-MTK-C** **F---------** **----------** **----------** **----------**

**RPW1_contig_14511** **ESWF--DGVA** **DKIVATCLKE** **AENATQYQVK** **PTSDNIKQCN** **PS--------** **--GITIKH-C** **LF--------** **-----REIQL** **SCPADQIKDQ** **KACDKFQDRI**

**RPW1_contig_16551** **PSDV------** **-DDITTCLSH** **VKD-------** **--MLI--TCN** **DI--------** **--KK-MLR-C** **FK--------** **----------** **-------LP-** **----------**

**RPW1_contig_1689**  **ETKV------** **DDILDTCYIH** **----------** **--YDT--PQE** **SA--------** **--FN-MMK-C** **MF--------** **---------K** **EH--------** **----------**

**RPW1_contig_17793** **NDKV------** **TAIMRKCFVQ** **----------** **--KST--AEE** **TA--------** **--LS-SLM-C** **FS--------** **---------E** **----------** **----------**

**RPW1_contig_19755** **GDKL------** **DKLLDRCYEK** **----------** **--HDT--QQE** **TA--------** **--FK-MMQ-C** **IF--------** **----------** **----------** **----------**

**RPW1_contig_2374**  **PDTV------** **HNLISACVQK** **----------** **--KDT--PEE** **TA--------** **--YQ-TFV-C** **YH--------** **---------E** **KNPNHSPIF-** **----------**

**RPW1_contig_257**  **NEDQ-----T** **KKLMEACAVT** **----------** **--NED--PKL** **QA--------** **--FN-AFK-C** **IH--------** **---------N** **KAKINLL---** **----------**

**RPW1_contig_29**  **--------IL** **MAALDKCKNI** **DG--------** **--GSD--LCE** **KA--------** **--YN-FNY-C** **LH--------** **---------Q** **ADPENWFLV-** **----------**

**RPW1_contig_3199**  **PEFM---NKL** **LSVQVTCGKI** **EL--------** **--DSD--PCE** **TA--------** **--LN-AAK-C** **AK--------** **---------E** **EGTKLGITSI** **----------**

**RPW1_contig_3213**  **EAD-------** **-PIIRSCGTK** **V---------** **--GKT--ACE** **NA--------** **--WL-THK-C** **YA--------** **---------E** **NFAEYMLI--** **----------**

**RPW1_contig_7073**  **PEQD------** **KDLIASCLGD** **----------** **--SKD--PEE** **IA--------** **--FH-TYI-C** **YY--------** **---------K** **ANP-------** **----------**

**RPW1_contig_8586**  **ESKV------** **SHILKDCAVA** **----------** **--KDT--PEQ** **TS--------** **--ID-LDA-C** **L---------** **----------** **----------** **----------**

**RPW1_contig_9136**  **PEAT------** **IELVTKCLGK** **----------** **--EGT--PED** **IA--------** **--FE-TFT-C** **YY--------** **---------K** **NNPNR-----** **----------**

**RPW1_contig_981**  **PDTV------** **HNLISACVQK** **----------** **--KDT--PEE** **TA--------** **--YQ-TFV-C** **YH--------** **---------E** **KNPKHSPIF-** **----------**

**RPW1_contig_9915**  **--------AV** **KKVAVDCDAL** **E---------** **--TKN--VSE** **KL--------** **--HK-FVV-C** **FI--------** **---------K** **TSPV------** **----------**

**RPW_OBP_Unigene_1** **----HNMENI** **HNIVKKCLIQ** **----------** **--KED--VLS** **TL--------** **--RA-AVQ-C** **FV--------** **---------N** **ESHNL-----** **----------**

**RPW_OBP_Unigene_2** **PDAV------** **NSLISTCVQS** **----------** **--KGS--PEE** **TA--------** **--FH-TFV-C** **YY--------** **---------E** **KTPTHVSIF-** **----------**

**RPW_OBP_Unigene_3** **AETV------** **NSLISACVQN** **----------** **--KGS--PEE** **TA--------** **--YQ-TFM-C** **YY--------** **---------E** **KTPTHASIF-** **----------**
