# Supplementary material for: Identification of the genes involved in odorant reception and detection in the palm weevil Rhynchophorus ferrugineus, an important quarantine pest, by antennal transcriptome analysis
Source: BMC Genomics. 2016 Jan 22;17:69. doi: 10.1186/s12864-016-2362-6 (PMC4722740; doi:10.1186/s12864-016-2362-6)
Supplement: Additional file 7: Figure S6 (A-F). — The relative abundances of different chemosensory gene families in the R. ferrugineus antennal transcriptome dataset, presented as reads per kilobase per million reads (RPKM). (DOCX 52 kb) [file 12864_2016_2362_MOESM7_ESM.docx]

**Additional file 7: Figure S6 (A-F). The relative abundances of different chemosensory gene families in the *R. ferrugineus* antennal transcriptome dataset, presented as reads per kilobase per million reads (RPKM).** The RPKM values were calculated for assembled contigs based on their mapping data according to the formula published in Mortazavi et al. [32]. The star sign (*) indicate highest RPKM value. The data presented here, did not include the biological replicates, hence further confirmation using qRT-PCR validation is recommended.

*****

*****

*****

*****

*****

*
