# Supplementary material for: Identification of the genes involved in odorant reception and detection in the palm weevil Rhynchophorus ferrugineus, an important quarantine pest, by antennal transcriptome analysis
Source: BMC Genomics. 2016 Jan 22;17:69. doi: 10.1186/s12864-016-2362-6 (PMC4722740; doi:10.1186/s12864-016-2362-6)
Supplement: Additional file 8: Figure S7. — A family of divergent odorant binding proteins (OBP) in R. ferrugineus. (DOCX 63 kb) [file 12864_2016_2362_MOESM8_ESM.docx]

**Additional file 8: Figure S7. A family of divergent odorant binding proteins (OBP) in *R. ferrugineus*.** An unrooted neighbor joining tree was constructed using an alignment of the *R. ferrugineus* OBPs using MEGA (v.6.0) [42] (JTT model for NJ heuristic searches methods with bootstrap support of 1000 replications). The RPW contigs colored with red bubble indicate the *R. ferrugenieus* OBPs not clustered with *B. mori* OBPs [50], form monophyletic group of coleopteran specific (except RPW_OBP_Unigene 1). *R.* *ferrugineus* OBPs: Minus-C, Plus-C, ABP I, ABP II, PBP/GOBP and CRLBP are indicated in blue, pink, green, orange, grey and yellow color respectively. Scale represent 0.2 amino acid substitution per site.

**CRLBP**

**Minu-C**

**Minu-C**

**CRLBP**

**ABP II**

**ABPII**

**CRLBP**

**CRLBP**

**ABPI**

**Minu-C**

**Plus-C**

**ABPII**

**ABPII**

**ABPII**

**Plus-C**
